# Carbohydrate Ingestion on Exercise Metabolism and Physical Performance

**DOI:** 10.1210/endrev/bnaf038

**Published:** 2026-01-21

**Authors:** Timothy D Noakes, Philip J Prins, Alex Buga, Dominic P D’Agostino, Jeff S Volek, Andrew P Koutnik

**Affiliations:** Emeritus Faculty of Health Sciences, University of Cape Town, Cape Town 7700, South Africa; Department of Exercise Physiology, Grove City College, Grove City, PA 16127, USA; Department of Human Sciences, Ohio State University, Columbus, OH 43210, USA; Department of Molecular Pharmacology and Physiology, University of South Florida Morsani College of Medicine, Tampa, FL 33602, USA; Human Healthspan, Resilience, and Performance, Florida Institute for Human and Machine Cognition, Pensacola, FL 32502, USA; Department of Human Sciences, Ohio State University, Columbus, OH 43210, USA; Human Healthspan, Resilience, and Performance, Florida Institute for Human and Machine Cognition, Pensacola, FL 32502, USA; Institute for Sport Science and Medicine, Department of Health, Nutrition, and Food Sciences, Anne's College of Education, Health, and Human Sciences, Florida State University, Tallahassee, FL 32506, USA

**Keywords:** carbohydrates, exercise, metabolism, performance, hypoglycemia, glycogen

## Abstract

Carbohydrate (CHO) ingestion during exercise has long been associated with improved performance. Early Scandinavian research proposed that CHO ingestion mitigates exercise-induced hypoglycemia (EIH) through a central neural mechanism, preventing glycopenic brain damage. Subsequent studies linked muscle glycogen depletion to fatigue during prolonged exercise, suggesting an obligatory reliance on glycogen, while overlooking the simultaneous presence of profound EIH at exhaustion. However, emerging evidence challenges this paradigm highlighting EIH role in fatigue. We comprehensively review more than 100 years of evidence from more than 160 studies looking at CHO ingestion, exercise metabolism, and physical performance that demonstrates the following key findings: (1) EIH correlates strongly with exercise termination, while muscle glycogen depletion alone does not induce rigor or whole-body fatigue; (2) CHO ingestion reduces liver glycogenolysis, preserves blood glucose, and paradoxically accelerates muscle glycogen breakdown through conserved neuroendocrine mechanisms; (3) high-fat-adapted athletes demonstrate exceptional fat oxidation, equivalent exercise performance, despite lower glycogen and CHO oxidation, challenging the belief that glycogen and CHO oxidation are central to exercise performance or that CHO is an obligatory fuel; and (4) CHO ingestion during exercise significantly enhances performance, even in glycogen-depleted states, by eliminating EIH. These data demonstrate that the main benefit of CHO ingestion before or during exercise is to prevent EIH, highlighted in prolonged efforts (>2-3 hours) and individuals with insufficient hepatic gluconeogenesis. This has important implications for sports dietary recommendations (ie, habitual high- or low-CHO diets) and the amount of CHOs athletes should be encouraged to ingest during exercise to maximize performance.

## Essential Points

- Reevaluation of EIH as a critical factor in fatigue: The manuscript provides the most comprehensive analyses of CHO administration to evaluate the long-held belief that muscle glycogen depletion is the primary cause of fatigue during prolonged exercise, suggesting instead that EIH, mediated by inadequate hepatic glucose production and falling blood glucose (BG) levels, is the central determinant
- Carbohydrate ingestion mitigates EIH: Evidence from more than 160 analyzed studies confirms that CHO ingestion during prolonged exercise prevents or reverses EIH, enabling sustained exercise performance by stabilizing BG levels rather than replenishing muscle glycogen
- Muscle glycogen depletion and energy crisis hypothesis disputed: This review disputes the traditional “energy crisis,” originally based on hypothesis and associative evidence, demonstrating that fatigue does not occur due to adenosine triphosphate (ATP) depletion or muscle rigor but as a brain-regulated protective mechanism to prevent hypoglycemic brain damage
- Role of hepatic glycogen in endurance performance: The findings emphasize the critical role of hepatic glycogen and glucose production in maintaining BG levels during prolonged exercise, highlighting its importance over skeletal muscle glycogen in endurance performance
- High-CHO diets and fat oxidation: Chronic adaptation to low-CHO diets leads to enhanced fat oxidation, challenging the belief that CHO is an obligatory fuel during high-intensity exercise and prolonged endurance efforts
- Efficacy of low-dose CHO supplementation: The study finds no dose-dependent improvement in exercise performance beyond low-dose CHO ingestion (∼15-30 g/h), emphasizing that the prevention of EIH is the primary benefit, irrespective of the quantity ingested
- Implications for athletic nutrition guidelines: The manuscript updates existing recommendations for high-CHO intake during exercise, as the evidence demonstrates that individualized approaches that prioritize BG stabilization over excessive CHO consumption are optimal and consistent with all available evidence

Needle biopsies were introduced in 1967 (1), enabling muscle and later liver glycogen concentrations to be measured before, during, and after exercise. This led to the theory that muscle glycogen plays a special, indeed obligatory role in human exercise metabolism. During high-intensity exercise at greater than 85% of the individual's maximum oxygen consumption (85%VO_2_max), muscle glycogenolysis provides the majority of energy for skeletal muscle contraction so that: “Glycogen is the primary fuel supporting ATP homeostasis during moderate-to-intense exercise (2)” (3), whereas during more prolonged exercise at a lower %VO_2_max, depletion of muscle glycogen content is the main cause of fatigue and exercise termination: “When the whole glycogen store is utilized the power output will decrease to a level where the energy demand rate can be met by oxidation of fat (4, 5) ie, work intensities demanding less than 65% of VO_2_max” (6).

Since this research also established that the dietary carbohydrate (CHO) intake in the days before exercise determined the preexercise muscle glycogen content, supporting advice that athletes should eat high CHOs to maximize their performances (7-9). This diverged from the traditional dietary advice for athletes (10). From 1896 to 1996, Olympic athletes preferred protein-rich foods. For example, at the 1960 Olympic Games “carbohydrate-based foods were not a catering priority” (10). Sixteen years later at the 1976 Montreal Olympic Games, the emphasis remained on a “high intake of protein” (11).

But by the 1996 Olympic Games in Atlanta, the shift to a high-CHO low-fat diet was complete: The official report from those Games reported that the diet provided was a “high-starch, low-fat menu” (12). Studies at the 2000 Sydney Olympic Games confirmed this change: “carbohydrate-rich foods were eaten at all meals, with cereal being the most popular at breakfast, and pasta, rice, and potato most popular at lunch and dinner … Data obtained on food consumption suggested this cohort of patrons was conscious of healthier food choices. Whole-grain breads and cereals were reported to be more popular than refined varieties and reduced-fat milk was consumed more than full-fat milk. Demand for carbohydrate-based options such as pizza, pasta, and rice outstripped catering's estimated projections … This demonstrates the move away from protein-based foods and towards an evidence-based higher-carbohydrate diet to optimize sports performance” (10 pp.328-329).

**Figure 1.**
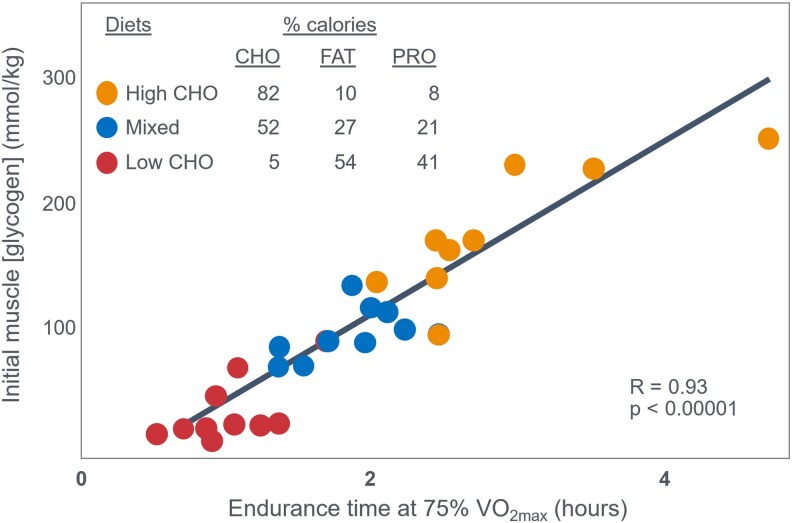
An apparent linear relationship between the preexercise glycogen concentration in the quadriceps femoris muscle and endurance time during exercise at 75% VO_2_max. Reproduced from (4).

This shift was driven by studies in the late 1960s (4, 13-25) reporting the results of studies using this novel biopsy technique. The single study most often cited as definitive proof for an obligatory role of muscle glycogenolysis in sustaining prolonged exercise performance reported that, when the preexercise was high in CHO so that individuals began exercise with elevated glycogen concentrations, they were able to sustain exercise at 75% VO_2_max for significantly longer than when they ate CHO-restricted diets and began exercise with lower glycogen concentrations (4) (Fig. 1).

**Figure 2.**
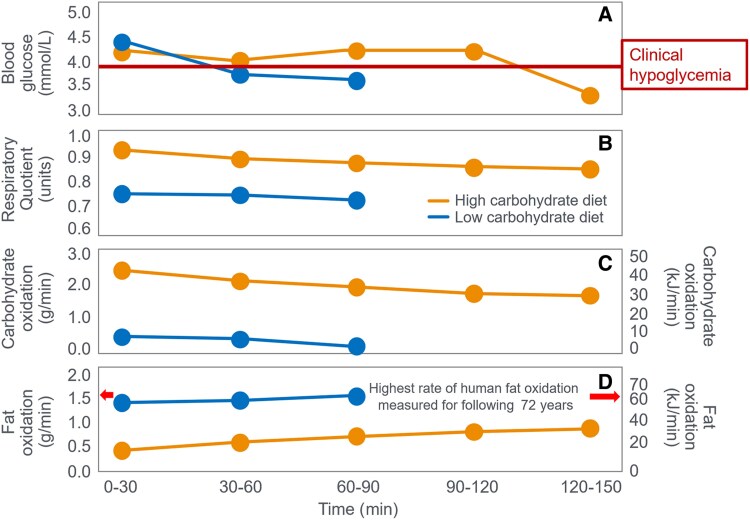
(A) Blood glucose concentrations, (B) respiratory quotient, and (C) rates of carbohydrate (CHO) and (D) fat oxidation in Ove Boje during 90 to 150 minutes of exercise when eating a preexercise diet either high or low in CHO. Both exercise bouts terminated with (A) very low blood glucose concentration (<3.65 mmol/L). Following the LCHO diet, Boje recorded the highest rates of (D) fat oxidation (1.51 g/min) measured in humans prior to 2015. Fat oxidation data calculated from respiratory quotient (RQ) and VO_2_ data reported in (27) using conventional equations. (A) A line indicating the blood glucose concentration at which clinical hypoglycemia is diagnosed is also included in this and all subsequent figures.

The authors concluded: “The good correlation between initial muscle glycogen concentration and work time (Fig. 2) demonstrates that the individual's ability to sustain prolonged exercise is highly dependent on the glycogen content of the muscles which, in turn, is dependent on the type of diet before exercise” (p. 148).

A study published 4 years later (21) seemed to confirm this conclusion. It found that some athletes who ate a high-CHO diet for 3 or more days before competition—so-called CHO-loading—finished a 30-km running race up to 15 minutes faster than when they ate a lower-CHO preexercise diet. Importantly, Karlsson and Saltin (21) did not report blood glucose (BG) concentrations in the runners in their 1971 trial. Nevertheless, this finding supported the conclusions of Krogh and Lindhard (26) nearly 50 years earlier that for some individuals “on (high) fat diets the fatigue (during exercise) became considerable and sometimes excessive. For several hours after the work on the ergometer these subjects were very tired when on a fat diet and much less tired or not tired at all when on carbohydrate.” It was also compatible with the findings of Boje (27) and Christensen and Hansen (28, 29) that individuals eating a low-CHO preexercise diet terminated exercise prematurely. For example, when Boje himself was the subject in Christensen and Hansen's original study (28), he developed premature hypoglycemia with an extraordinarily low rate of CHO oxidation (see Fig. 2) after just 90 minutes of exercise when eating a low-CHO preexercise diet. His peak rate of fat oxidation (1.51 g/min) was also extraordinarily high and surpassed only by athletes participating in studies published more than 80 years later (30, 31). As a result, during the 90 minutes that Boje exercised following the low-CHO diet, approximately 83% of his energy came from the oxidation of fat.

Several mechanisms may explain the reliance on muscle glycogen during prolonged exercise. The most popular is the anaplerotic theory (TAT) (6, 32-37) also called the energy crisis hypothesis (38), perhaps most clearly stated by Fitts (34) as it relates to the effects of CHO ingestion in delaying or reversing fatigue during more prolonged exercise: “… the preponderance of evidence supports the notion that CHO ingestion delays fatigue by maintaining a high CHO fuel source in the form of blood glucose (39, 40).” This implies that CHO oxidation is essential to the maintenance of prolonged exercise at moderate to high intensity (65%-90% of one's maximal oxygen uptake [VO_2_max]). To date, a cellular mechanism for the obligatory oxidation of CHO has not been established (present authors' emphasis). The possibilities include the following:

1. High muscle oxidation rates cannot be maintained without a CHO fuel source
2. CHO supply critical metabolic intermediates so that CHO depletion reduces the oxidation rate of available fats and proteins, or
3. CHO depletion is correlated with (and causative of) changes in other cellular events that in turn elicit fatigue. *Little direct support exists for any of these possibilities* (present authors' emphasis)” (34).

Others (37, 41-45) associate glycogen depletion with impaired Ca2+ release or Na+–K+ ATPase activity (46, 47), though these links remain unclear (41) or still to be established (37, 43). Jensen et al (48) suggest there is heterogeneity in skeletal muscle glycogen depletion patterns and that the intramyofibrillar glycogen pool appears to be the most important determinant of prolonged exercise performance. Nielsen et al (49)) proposed that skeletal muscle fibers with higher oxidative capacity are the first to become glycogen depleted so that, as prolonged exercise continues, skeletal muscle fibers with less oxidative capacity must be recruited, causing a reduction in exercise capacity. Others suggest (50) that increased fat oxidation from low-CHO high-fat diets may raise energy cost and impair movement economy, reducing performance (50-55). Cross-bridge cycle studies now offer additional insights into fatigue mechanisms (56). Still, liver glycogen—also boosted by CHO-loading (20, 23)—may delay EIH (30, 57), as seen in Boje (see Fig. 2) (27).

**Figure 3.**
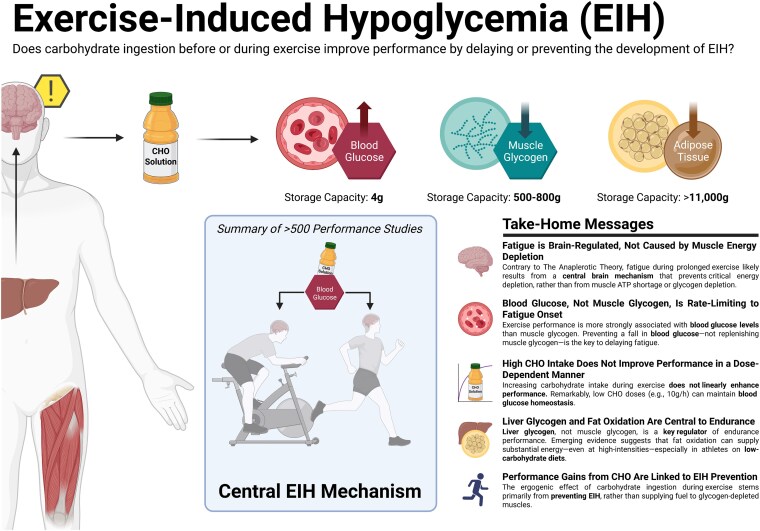
Carbohydrate (CHO) ingestion during exercise has long been associated with improved performance. Early Scandinavian research proposed that CHO ingestion mitigates exercise-induced hypoglycemia (EIH) through a central neural mechanism, preventing glycopenic brain damage. Subsequent studies linked muscle glycogen depletion to fatigue during prolonged exercise, suggesting an obligatory reliance on glycogen, while overlooking the simultaneous presence of profound EIH at exhaustion. However, emerging evidence challenges this paradigm highlighting EIH role in fatigue. We comprehensively review more than 100 years of evidence from more than 160 studies looking at carbohydrate ingestion, exercise metabolism, and physical performance that demonstrate the following key findings: (1) EIH correlates strongly with exercise termination, while muscle glycogen depletion alone does not induce rigor or whole-body fatigue; (2) CHO ingestion reduces liver glycogenolysis, preserves blood glucose, and paradoxically accelerates muscle glycogen breakdown through conserved neuroendocrine mechanisms; (3) high-fat–adapted athletes demonstrate exceptional fat oxidation, equivalent to exercise performance, despite lower glycogen and CHO oxidation, challenging the belief that glycogen and CHO oxidation are central to exercise performance or that CHO is an obligatory fuel; and (4) CHO ingestion during exercise significantly enhances performance, even in glycogen-depleted states, by eliminating EIH. These data demonstrate that the main benefit of CHO ingestion before or during exercise is to prevent EIH, highlighted in prolonged efforts (>2-3 hours) and individuals with insufficient hepatic gluconeogenesis. This has important implications for sports dietary recommendations (ie, habitual high- or low-CHO diets) and the amount of CHOs athletes should be encouraged to ingest during exercise to maximize performance.

In this manuscript, all data demonstrate that the main benefit of CHO ingestion before or during exercise is to prevent the development of EIH, which appears inevitable if the exercise is prolonged for more than 2 to 3 hours and is undertaken by individuals who are unable to increase hepatic gluconeogenesis sufficiently (Fig. 3), as outlined later across 16 different topics. Accordingly, this has important implications for advice on the value of habitual high- or low-CHO diets for athletes as well as the amount of CHO athletes should be encouraged to ingest during exercise to maximize performance. The data reviewed herein present the novel interpretation that nutritional strategies to maximize performance during prolonged exercise should be geared to maintaining the small glucose pool (SGP) during exercise rather than filling (or overfilling) the large glucose pool (LGP) before exercise. The present evidence indicates that this can be achieved by ingesting relatively small amounts of CHO (∼10 g/h) during exercise. This review outlines the following key points:

1. The mechanism of depletion of the skeletal muscle LGP causes fatigue remains hypothetical. The usual explanation is the TAT, which postulates that muscle glycogen depletion during exercise causes an inevitable “energy crisis.” But this is implausible since a developing “energy crisis” must produce skeletal muscle ATP depletion leading ultimately to muscle rigor, not whole-body fatigue. But this does not happen. A more probable explanation is that exercise fatigue is a brain-control mechanism designed to cause exercise termination before irreversible energy depletion occurs in the exercising skeletal muscles.
2. An iconic 1967 Scandinavian study failed to acknowledge that individuals eating CHO-restricted diets terminated exercise, not with low muscle glycogen content alone, but also with marked EIH.
3. Early studies by Boje, Christensen, and Hansen showing that CHO ingestion could reverse exhaustion in individuals during prolonged exercise concluded that: “Fatigue must be regarded as a hypoglycemic symptom of cerebral origin.”
4. Series of carefully conducted studies in the 1980s concluded that Boje, Christensen, and Hansen's interpretation was incorrect, postulating rather that CHO ingestion prolongs exercise, not by preventing or reversing EIH, but by providing an obligatory (exogenous) source of CHO for muscles that have become glycogen depleted during prolonged exercise. However, none of these new studies provided definitive evidence to support this replacement hypothesis, which currently remains unproven and largely untested with little or no experimental support.
5. Eighty-eight percent of studies that reported a clear benefit on exercise performance of CHO ingestion before or during exercise also found that BG concentrations fell during exercise in the control (placebo) group. This is important since few if any of these studies were actively studying a falling BG concentration as an important factor causing exercise fatigue.
6. The neural mechanisms by which falling BG concentrations limit the recruitment of skeletal muscle motor units to prevent the risk of glycopenic brain damage is well described. The biological effect of hypoglycemia on exercise performance must be considered relative to an individual's baseline glucose levels and the magnitude of glucose reduction, as counterregulatory responses and symptoms vary by person, can occur subperceptually, and may manifest at higher glucose levels in those with chronically elevated glycemia. Consequently, the “drop” in BG relative to a prior value has important biological consequences in exercise performance beyond the absolute value of BG.
7. Increasing the amount of CHO ingested during exercise increases the rate of exogenous CHO oxidation. According to the novel replacement theory, this additional source of obligatory CHO should produce an easily detectable dose-dependent increase in exercise performance. However, studies of progressively higher rates of CHO ingestion during prolonged exercise fail to show serial dose-dependent improvements in exercise performance. Rather the lowest CHO dose that is tested usually produces ergogenic effects equivalent to those produced by much higher rates of CHO ingestion. This effect is most logically explained by CHO acting to prevent depletion of the SGP in the blood and liver, rather than by depletion of the LGP in skeletal muscle.
8. The novel replacement hypothesis has produced expert guidelines encouraging athletes to ingest up to 2-g CHO/min during prolonged exercise to prevent depletion of the LGP. Illogically, these guidelines are also promoted to athletes participating in exercise lasting more than 3 hours. Yet, prolonged strenuous exercise (eg, ultramarathon races) are often completed at exercise intensities (60%-75%VO_2_max) at which intensities fat oxidation should be able to provide a substantial proportion of the required energy, especially as rates of fat oxidation increase with progressive muscle glycogen depletion (see also topic 15).
9. Muscle glycogen is not “spared” during prolonged exercise as would be expected if the LGP is the cardinal driver of exercise performance. Instead, the rate of muscle glycogen use during exercise is set by the muscle glycogen concentration at the start of exercise. Paradoxically high rates of CHO ingestion during exercise increase muscle glycogenolysis.
10. This is because high rates of CHO ingestion or infusion during exercise reduce rates of fat oxidation. This would explain the increased rates of muscle glycogenolysis with high rates of CHO ingestion or infusion during exercise. In effect, muscle glycogen determines its rate of use during exercise by setting the rate at which the major alternate fuel, fat, is oxidized. This effect appears to be hormonally regulated.
11. In contrast, CHO ingestion during exercise reduces or, at high rates of intake, completely suppresses liver glycogenolysis. This effect is the result of control mechanisms aimed at maintaining a stable BG concentration, which is considered the principal aim of human metabolism.
12. During prolonged exercise, whole-body CHO oxidation decreases, while BG oxidation progressively increases, regardless of intensity. Consequently, EIH is probable without CHO intake, particularly when starting with low liver glycogen and limited hepatic gluconeogenesis capacity.
13. Falling liver glycogen concentrations stimulate adipose tissue lipolysis, increasing muscle fat oxidation, further sparing both BG and muscle glycogen use.
14. Rats that overexpress the protein targeting to glycogen (PTG) have elevated liver glycogen content at rest and during both exercise and fasting. They also have greater resistance to EIH and superior exercise performance. Rats with a greater capacity to store muscle glycogen do not have superior exercise performance, nor do rats unable to synthesize muscle glycogen have impaired exercise performance. The authors conclude that: “…these results identify hepatic glycogen as a key regulator of endurance performance in mice, an effect that may be exerted by the maintenance of blood glucose concentrations.”
15. Recent studies establish that athletes chronically adapted to low-CHO diets achieve the highest rates of fat oxidation yet measured in humans (>1.5 g/min) even when exercising at more than 85% of their VO_2_max. This challenges the existence of the exercise cross-over point, which holds that CHO oxidation increases and fat oxidation decreases with increasing exercise intensity so that above approximately 85% VO_2_max only CHO is oxidized. Rather, this finding suggests that fat oxidation fuels exercise even during high-intensity exercise. This in turn challenges the concept that CHO is an obligatory fuel both for high-intensity and prolonged exercise.
16. A recent study found that the performance of athletes habituated for 6 weeks to either high- (380-g CHO/d) or low- (40-g CHO/d) CHO diets was not different during prolonged submaximal exercise, despite the low-CHO diet having lower glycogen and CHO oxidation levels. However, ingesting 10-g CHO/h during exercise increased exercise performance by 12% to 20% while eliminating EIH. As glycogen and whole-body CHO were lower on the low-CHO diet across multiple analyses, yet performance was maintained. This directly challenges glycogen and whole-body CHO oxidation and instead suggests EIH is a central determinant of exercise performance.

**Developing an alternative hypothesis: TAT is implausible and has been disproven. Liver (not muscle) glycogen depletion leading to a falling BG concentration**—**EIH—activates a protective neural reflex to limit prolonged exercise performance specifically to prevent glycopenic brain damage.**

## Evidence 1: TAT is Biologically Implausible and Disproven

TAT suggests muscle ATP must fall drastically at exhaustion, especially with low glycogen. But this is illogical—if ATP fell too low, muscle rigor would occur (57-59), which is never seen. Likely, another mechanism—perhaps brain-regulated (60)—prevents ATP depletion, aided by peripheral signals (61, 62), even as ATP use spikes 600-fold during intense exercise (63). At least 10 studies show muscle ATP does not fall at fatigue in prolonged (64-75) or high-intensity exercise (76)). Another found low-CHO diets reduce pyruvate dehydrogenase activity and glycogenolysis without impairing ATP or performance (77) A third found high-intensity glycolysis unaffected by glycogen depletion (78). In summary, ATP depletion would cause rigor (58, 60), not fatigue (57, 59). Since no rigor cases exist in the scientific literature, TAT is invalid.

As Green concluded in 1991: “Although it has been popular to implicate a compromised energy status, occurring as the result of the depletion of glycogen in the muscle fiber, such a proposition is not supported by present experimental evidence” (38 p.295). Fifty-six years after the foundational study (4), the mechanism linking glycogen depletion to contractile failure remains unknown—if real, it should be well established by now.

## Evidence 2: The Original Scandinavian Study Linking Muscle Glycogen and Exercise Duration Ignored Key Findings of EIH and High-fat Oxidation in CHO-restricted Individuals

**Figure 4.**
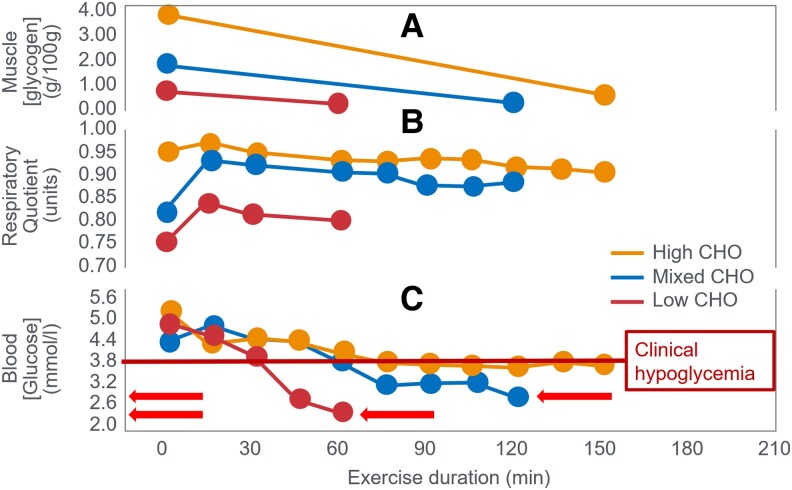
Changes in (A) muscle glycogen concentration, (B) respiratory quotient, and (C) blood glucose concentrations during prolonged submaximal exercise at 75% VO_2_max in individuals who had eaten preexercise diets either high, mixed, or low in carbohydrates (CHO). Note development of a progressive, marked exercise-induced hypoglycemia following the 2 acute diets lowest in CHO (arrows) (4). This latter finding has escaped critical interpretation for 55 years (57).

The iconic Scandinavian study (4) linked muscle glycogen to exercise duration at 75% VO_2_max and tracked BG levels, revealing severe EIH in low-CHO trials (arrows in Fig. 4) and elevated fat oxidation, as originally shown by Boje (see Fig. 2)—patterns overlooked for 56 years.

Despite long-standing knowledge that EIH impairs performance, and its reversal extends performance (27, 29), this has been ignored for 55 years (30, 57, 59) (see next section). The authors cited Christensen and Hansen's (28) finding: Low-CHO diets cut endurance from 240 to 90 minutes. Yet they omitted the follow-up (29), showing CHO intake reversed EIH and restored performance. They noted that BG “fell to extremely low levels after the protein diet, causing general fatigue, headaches, and dizziness… possibly due to liver glycogen depletion” (p. 149). However, they failed to acknowledge the low BG role in central fatigue, as Christensen and Hansen did. In 1986, Hultman (4) acknowledged body fat is not limiting in exercise performance—rather, liver glycogen depletion lowers BG, causing central fatigue. Low-CHO diets impair liver glucose output, increasing EIH risk (2). One can retrospectively analyze the 1967 data (4) to assess whether muscle glycogen depletion or EIH caused fatigue across preexercise diet types (Table 1).

**Table 1.** Metabolic variables at the point of fatigue in 9 individuals exercising for different durations after they had eaten 3 diets different in their carbohydrate contents for the final 3 days before exercise

Variable	High CHO diet	Moderate CHO diet	Low CHO, fat protein diet	Difference: high CHO minus low CHO, fat protein diet (units) (%)
VO_2_ (l/min)	3.16	3.24	3.17	−0.01 (−0.32%)
RQ (units)	0.918	0.882	0.795	0.123 (56%)
Rate of CHO oxidation (g/min) (kJ/min) at fatigue	2.80 47.6	2.43 41.3	1.24 21.1	1.56 (56%) 26.5 (56%)
Rate of fat oxidation (g/min) (kJ/min) at exhaustion	0.48 18.2	0.70 26.6	1.20 45.6	−0.72 (−150%) 27.4. (−150%)
Blood (glucose) (mmol/L) at exhaustion	3.52	2.99	2.82	0.70 (19.9%)
Muscle (glycogen) (g/100 g muscle) at exhaustion	0.43	0.17	0.13	0.30 (70%)
Exercise time to exhaustion, min	166.5	113.6	56.9	109.6 (193%)

Data from reference (4).

Abbreviations: CHO, carbohydrate; RQ, respiratory quotient; VO_2_max, maximum oxygen consumption.

This analysis shows large variations across 3 different diets in glycogen (70%), respiratory quotient (RQ) (56%), CHO oxidation (56%), and fat oxidation (150%)—incompatible with a singular theory that (i) glycogen concentrations or (ii) CHO oxidation rates alone explain fatigue. In contrast, BG levels at exhaustion were similar (19.9% variance) despite a 109% difference in time to fatigue. The high-CHO diet increased CHO oxidation (26.5 kJ/min), offsetting a nearly equal drop in fat oxidation (27.4 kJ/min). BG was the most consistent metabolic factor at exhaustion across diets, unlike glycogen or oxidation rates (50%-150% variance across diets). This suggests low BG—EIH—was the trigger for fatigue in this iconic trial, not glycogen depletion or substrate oxidation rate. This iconic study supports the older theory (27-29), that falling BG signals the brain to terminate exercise.

## Evidence 3: 1930s Scandinavian Studies Showed CHO Ingestion at Exhaustion Reverses EIH and Fatigue, Without Immediately Reversing Muscle Glycogen Depletion or RQ (CHO Oxidation Rates)

**Figure 5.**
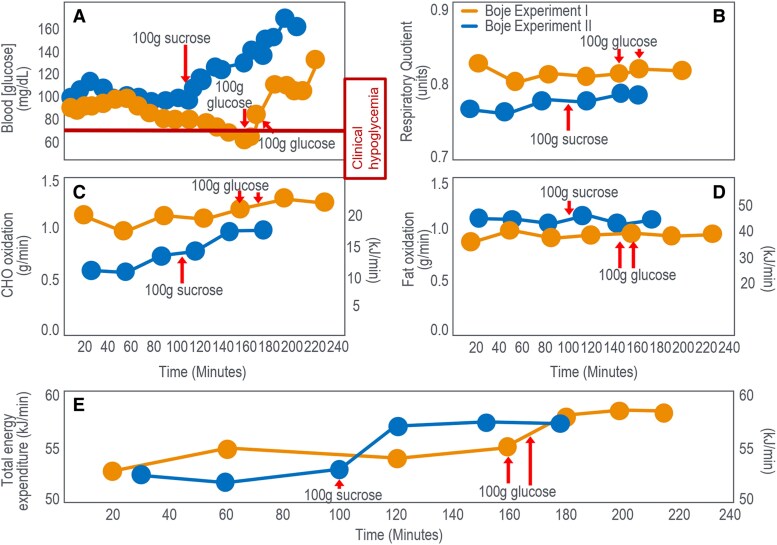
(A) Blood glucose concentration, (B) respiratory quotient (RQ), (C) rates of carbohydrate (CHO) and (D) fat oxidation, and (E) total energy expenditure in Boje in 2 separate experiments when he ingested CHO as he developed exhaustion. Rates of CHO and fat oxidation and total energy expenditure were calculated from the data for RQ and oxygen consumption (VO_2_) provided in the original paper (27).

In 1936, Boje (27) showed CHO ingestion at exhaustion reversed EIH. Fig. 5 shows 2 trials in which Boje was the participant.

In experiment I, Boje began tiring at 150 minutes with trouble continuing at 162 minutes. After ingesting 100 g glucose at 162 and 177 minutes (Fig. 5A), he recovered within 10 minutes and exercised 63 minutes more without symptoms. Boje reported, “he could have exercised even longer.” In experiment II, Boje took 100 g sucrose after 106 minutes and exercised to 210 minutes with distress (see Fig. 5A). A second participant had identical results (data not shown). The main effect of CHO ingestions across both experiments was EIH reversal (see Fig. 5A), with minimal effect on CHO (Fig. 5C) or fat oxidation (Fig. 5D), despite higher energy expenditure (Fig. 5E).

Boje (27) concluded that if exercise was sufficiently long, BG would fall (<60 mg/dL; 3.33 mmol/L), causing symptoms that blocked further exercise. The first feelings of exhaustion occurred concurrently with the greatest fall in blood “sugar” and “is intensified in parallel with this (fall)…. there was a parallelism between the subjective condition of the subject and the course of the blood sugar curve.” Symptoms reversed within 15 to 30 minutes of glucose or sucrose ingestion, allowing another hour of exercise, “just by taking sugar.” He also noted that a high-CHO diet 2 to 3 days prior to exercise extended exercise 2 to 3 times compared to high-fat diets. Finally, Boje noted that his RQ did not change as the blood “sugar” concentration rose and his symptoms disappeared (see Fig. 5B). He concluded that “one can see that the beneficial effect (of carbohydrate ingestion) is not based on a metabolic effect but probably comes about through an effect on the nervous system. Apparently, the nervous system requires a certain concentration of blood sugar to function normally.” Boje concluded the benefit came from restoring BG to support brain function. Fig. 5 shows that while the RQ remained unchanged, Boje increased his work output by approximately 7% after CHO ingestion in both experiments. As a result, his rate of CHO oxidation increased slightly (maximum increase 0.29 g/min) (see Fig. 5C), whereas his rate of fat oxidation varied little (see Fig. 5D), failing to increase, as a result of falling muscle glycogen and blood insulin concentrations (79, 80) as would be expected during exercise of this prolonged duration (see Fig. 2D).

CHO ingestion mainly raised BG without substantially changing CHO or fat oxidation (shown later). At exhaustion, fat was the dominant fuel—providing 37 kJ/min and 42 kJ/min in experiments 1 and 11 (see Fig. 2D), vs only 21 kJ/min and 17 kJ/min from carbohydrates (see Fig. 2C)—a pattern seen in a large number of studies (presented subsequently). It is understandable that, fixated on the belief that muscle glycogen is the obligatory fuel for prolonged exercise performance (see Fig. 1), no one has proposed that fatigue may stem from a failure to increase fat oxidation during prolonged exercise—even though fat supplied more energy at exhaustion in Boje’s trial (27) (see Fig. 2C and 2D).

**Figure 6.**
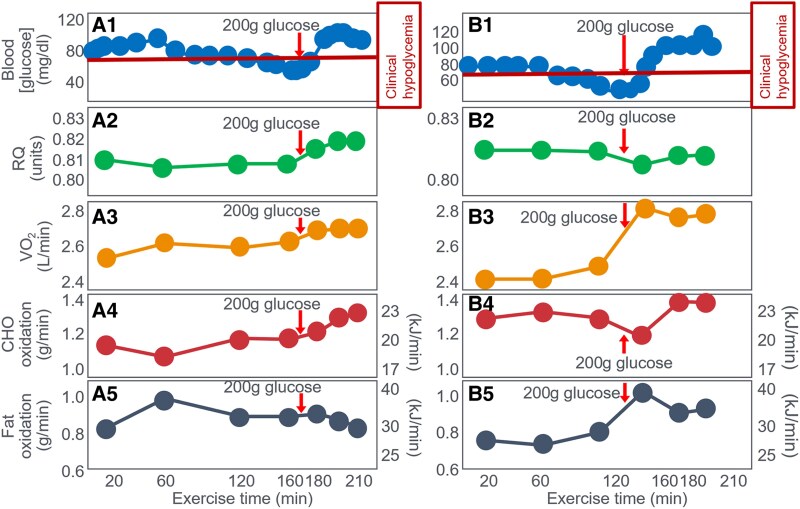
Changes in (A1 and B1) blood glucose concentrations, (A2 and B2) respiratory quotient (RQ), (A3 and B3) oxygen consumption (VO_2_) and rates of (A4 and B4) carbohydrate (CHO) and (A5 and B5) fat oxidation in 2 athletes (Athlete 1: A; Athlete 2: B) during prolonged exercise before and after they ingested 200 g glucose (arrows). Data for CHO and fat oxidation were calculated from VO_2_ and RQ (28, 29) using conventional equations.

In 1939 Christensen and Hansen (29) confirmed Boje's findings. CHO at fatigue reversed EIH and extended effort by 60 minutes, with minor RQ changes (27), which including Boje himself, showed that glucose ingested at the point of fatigue again reversed the EIH and the fatigue, allowing exercise to continue for another 60 minutes with a fall in RQ in one individual (Fig. 6B2) and a marginal increase in the other (Fig. 6A2). However, both participants increased their work outputs, showing an increase in VO_2_ after CHO ingestion (Fig. 6A3 and 6B3). As a result, there was a slight increase in the rates of CHO oxidation in both participants (0.17 and 0.08 g/min) (Fig. 6A4 and 6B4). The rate of fat oxidation was unchanged in one participant but initially increased somewhat (0.22 g/min) in the other (Fig. 6A5 and 6B5, respectively). Once again fat oxidation produced more energy at exhaustion (30 and 36 kJ/min) (see Fig. 6A5 and 6B5) than did CHO oxidation (23 and 23 kJ/min) (Fig. 6A4 and 6B4).

Christensen and Hansen (29) concluded: “The experimental data have shown an important independence between the RQ and the blood glucose values; even one hour after the administration of sugar during exercise, there was hardly any detectable increase in the RQ value even though the blood sugar had reached its maximum values already after about 30 minutes” (29 p.176). Thus, Christensen and Hansen (29) concluded BG—not glycogen or CHO burning—influenced recovery. CHO helped only if it raised BG. Fatigue was a cerebral hypoglycemic symptom. Together, these studies showed CHO ingestion at exhaustion prolonged exercise mainly by reversing EIH and prolonged exercise, not by boosting fuel oxidation (27, 29) (see Figs. 4 and 5).

Norwegian physiologist E.D.R. Pruett (81) supported this in the 1970s, in 72 work trials during which 9 individuals exercised to exhaustion at 50% or 70% VO_2_max while their BG concentrations were measured. All experiments at 50% VO_2_max and 15 of the 18 experiments at 70% VO_2_max terminated when BG concentrations fell to or below 3.0 mmol/L with central nervous system hypoglycemia symptoms. Symptoms faded approximately 15 minutes post exercise, as BG rose. Pruett concluded falling BG likely forced exercise termination. Using Hultman's data (19), Pruett estimated hepatic glucose production could rise to 1 g/min and account for 50% of total whole-body CHO oxidation. Since liver glycogen (50-100 g) could deplete over hours of exercise if not replenished, this explained BG drops and subsequent fatigue (p. 206).

## Evidence 4: Studies from the 1970s Revised the Explanation of How CHO Ingestion Prevents Fatigue, Shifting from a Central Brain Effect to Obligatory CHO Oxidation in Glycogen-depleted Muscles

**Figure 7.**
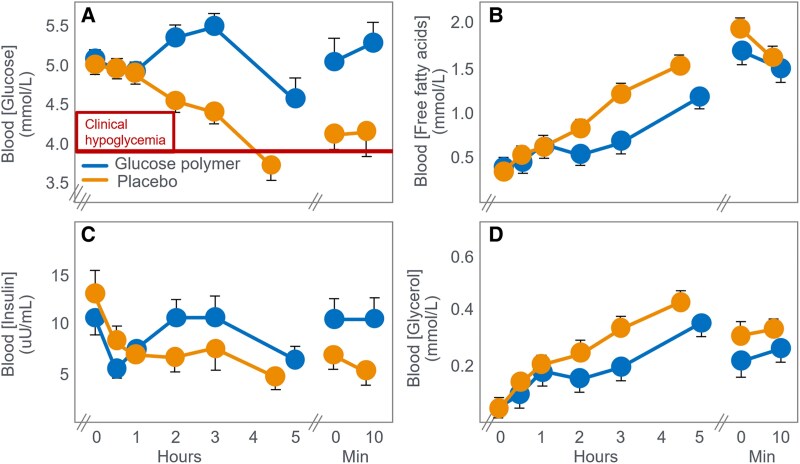
(A) Blood glucose, (B) free fatty acid, (C) insulin, and (D) glycerol concentrations during prolonged exercise (4-5 hours) at low intensity (45% VO_2_max) in individuals when they ingested either placebo or a glucose polymer (30 g/h). Reproduced from (86).

Following these original Scandinavian studies, a renewed interest in this topic began 37 years later, perhaps reawakened by the seminal presentation by John Wahren at the 1976 New York City Marathon Conference on the Marathon (82). That presentation emphasized their earlier finding (83) that during exercise of as little as 40 minutes, leg muscle glucose uptake exceeded the rate of hepatic glucose production so that: “(a) blood glucose becomes an increasingly important substrate for muscle oxidation during prolonged exercise of this type; (b) peripheral glucose utilisation increases in exercise despite a reduction in circulating insulin levels; (c) increased hepatic output of glucose, primarily by means of augmented glycogenolysis, contributes to blood glucose homeostasis in exercise and provides an important source of substrate for exercising muscle.” (p. 2715). As a result: “Mild hypoglycemia may subsequently develop and leg uptake of glucose declines slightly” (82 p.48). Ahlborg and Felig (84) described the effects of glucose ingestion (200 g) during exercise on the fuel-hormone response during low-intensity exercise (30% VO_2_max) lasting 4 hours. Glucose ingestion doubled splanchnic glucose output; doubled glucose uptake by the exercising muscles, rising to account for 60% of leg oxygen uptake; partially inhibited fat oxidation; and prevented the development of EIH. However, they calculated that 58% of the ingested glucose load was retained within the splanchnic bed, escaping oxidation. Two of the next studies were performed by Ivy and colleagues (85, 86). In their second study, Ivy et al (86) studied 10 trained male participants who walked at 45% VO_2_max for 4 to 5 hours on a laboratory treadmill while ingesting either a placebo or 30 g/h of a CHO solution. EIH began to develop in the placebo group after the first hour of exercise,e with BG concentrations reaching 3.7 mmol/L at premature exercise termination, 210 minutes later (Fig. 7A).

In their introduction to the study, the authors acknowledged the work of Christensen and Hansen: “…hypoglycemia causes central nervous system dysfunction and this manifests itself as physical exhaustion (28, 29)” (86 p.466). However, since psychometric testing did not reveal mental impairments in their participants even when they developed profound EIH, the authors concluded that: “In summary, these data suggest that exhaustion was not a result of hypoglycemia or central nervous system dysfunction. More importantly, however, was the finding that endurance capacity was enhanced by the CHO supplements taken during the walking exercise” (p. 470). The authors proposed unmeasured glycogen depletion, rather than documented EIH, caused premature fatigue—still the dominant theory. Importantly, the authors proposed that muscle glycogen depletion (which they did not measure), not EIH (which they did document), was the cause of the premature fatigue in the placebo group. This was, and remains, the dominant belief of the obligatory role of muscle glycogen depletion in determining fatigue during prolonged exercise. The authors did report the rates of energy expenditure and CHO and fat oxidation at the point of fatigue in the placebo and CHO-supplemented groups (Table 2). These data can also be examined retrospectively to determine whether they support the authors' conclusions. Importantly, this exercise bout was conducted at 45% VO_2_max, an exercise intensity at which CHO oxidation has been argued for decades to not be an essential energy source (5, 87-94). Recall the statement by Hultman et al in 1986 that at work intensities less than 65% of VO_2_max, the whole energy demand can be met by the oxidation of fat (6 p.S99).

**Table 2.** Rates of oxygen consumption (VO_2_) and whole-body carbohydrate and fat oxidation at fatigue in individuals ingesting either a placebo or a glucose-polymer supplement during 4 to 5 hours of treadmill walking at 45% VO_2_max

	Placebo control	CHO supplementation	Difference (CHO – placebo control)
VO_2_max (L/min)	^|^---- 4.3 ± 0.5 ----^|^		
VO_2_ (L/min)	2.02	2.16	0.14
Energy expenditure (kJ/min)	42.40	45.40	3.00
Respiratory quotient (units)	0.84	0.88	0.04
CHO oxidation (g/min) (kJ/min)	1.17 19.90	1.60 27.20	0.43 7.30
CHO ingestion rate (g/min) (kJ/min)	0.0 0.0	0.5 8.5	0.5 8.5
Fat oxidation (g/min) (kJ/min)	0.59 22.40	0.47 17.90	−0.12 −4.60
Blood glucose concentration (mmol/L)	3.70	4.60	0.90

Abbreviations: CHO, carbohydrate; RQ, respiratory quotient; VO_2_max, maximum oxygen consumption.

CHO ingestion increased CHO oxidation by 0.43 g/min (7.3 kJ/min), matching intake, and decreased fat oxidation by 0.12 g/min (4.6 kJ/min). This raises the relevant question: What specific metabolic advantage was provided by this 0.43 g/min of additional CHO oxidation? Why could fat oxidation at an extra rate of 0.18 g/min not substitute for that trivial amount of extra energy provided by CHO oxidation, especially during low-intensity exercise at 45% VO_2_max, an intensity at which fat oxidation can easily substitute for CHO oxidation? Perhaps the most logical explanation is that CHO ingestion improved performance by preventing EIH (see Fig. 7A). At the same time, the ingested CHO increased whole-body CHO oxidation to substitute for the reduced rate of fat oxidation produced by higher blood insulin and lower blood-free fatty acid concentrations (Fig. 7B and 7C). In retrospect, the following question arises: Why were the authors so convinced that CHO metabolism was more important than fat metabolism to sustain exercise at such a low exercise intensity? Especially when their data showed that, at exercise termination, the rate of fat oxidation (see Table 2) was 26% higher when participants ingested the placebo rather than CHO. This early sports drink–funded study may have biased authors toward emphasizing CHO over fat metabolism.

**Figure 8.**
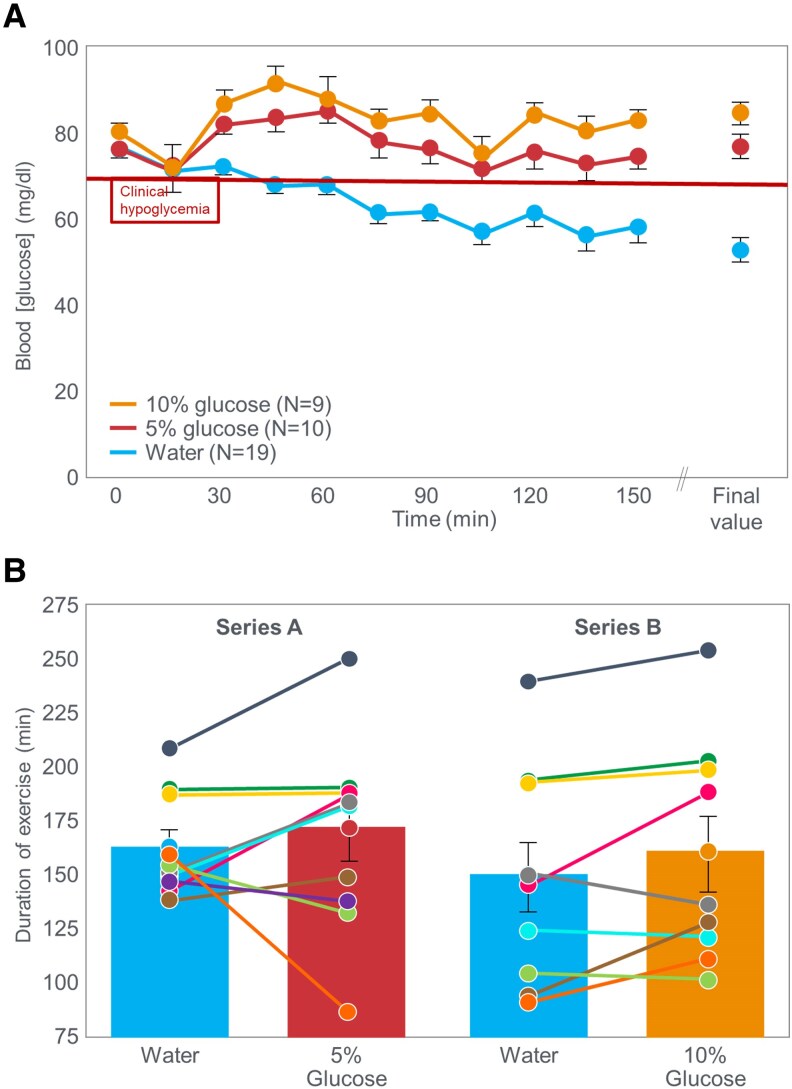
A, Blood glucose concentrations during exercise to exhaustion at 60% to 65% VO_2_max in healthy men aged 18 to 47 years when they ingested either placebo or a 5% or 10% glucose solution. B shows exercise time to exhaustion when individuals ingested either the 5% or 10% glucose solutions compared to exercise performance when ingesting placebo. Reproduced from reference (95).

Another important factor likely influencing this interpretation was a publication the previous year in *The New England Journal of Medicine* by Felig and Wahren (95), whose publications during the previous decade (82-84) had been so influential. Felig et al (95) exercised 19 “healthy men aged 18 to 47 years” at 60% to 65% VO_2_max until they were unable to sustain the necessary work rate. In one experiment, participants ingested either a CHO solution (5% or 10% glucose) providing either 40 or 80 g glucose/h, or a sweetened placebo. Fig. 8A shows that when ingesting the placebo, participants developed a progressive EIH starting after the first 30 minutes of exercise. Glucose ingestion at 80 g glucose/h prevented any decline in the BG concentration whereas ingestion at a rate of 40 g/h did not prevent a small drop in the BG concentrations after 75 minutes. Fig. 8B compares the exercise performances of individuals when they ingested either glucose or placebo. The authors concluded hypoglycemia did not affect endurance or consistently delay exhaustion (95). Yet, retrospectively, 13 of 19 participants improved or maintained performance with CHO, also reducing hypoglycemia. Also, this inherent participant variability suggests that the sample size for this experiment was perhaps too small to draw any definitive conclusions within this variable subject cohort. These 2 studies (86, 95) effectively erased Boje, Christensen, and Hansen's definitive findings (27-29, 96) from scientific memory. The original German publications may have been overlooked by English-speaking scientists in the 1980s.

**Figure 9.**
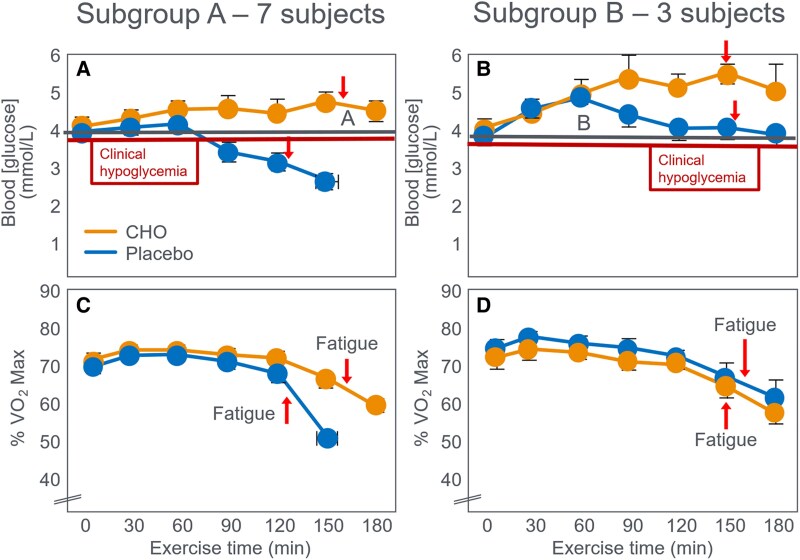
(A and B) Blood glucose concentrations and (C and D) exercise intensity (% VO_2_max) in 2 groups of participants during prolonged exercise. Seven individuals in subgroup A (A and C) developed progressive exercise-induced hypoglycemia (EIH) during exercise when ingesting placebo whereas 3 individuals in subgroup B (B and D) did not develop EIH when ingesting placebo. Carbohydrate (CHO) ingestion during exercise prevented EIH in all participants (CHO ingestion in A and B). (C) Fatigue developed prematurely in subjects who developed EIH during exercise. (D) No premature fatigue in subjects who mainted BG concentrations during exercise. Reproduced from (97).

In 1983, Coyle et al (97) published a study of 10 trained individuals who began exercise at 74% VO_2_max and continued for as long as they could sustain an exercise intensity greater than approximately 64% VO_2_max. In one trial, participants received a CHO drink; in the second, a placebo. In 7 of 10 participants (subgroup A), BG concentrations fell below 3.0 mmol/L 1 hour after starting placebo, causing early fatigue (Fig. 9A and 9C). The 3 remaining participants whose BG concentrations did not fall below the starting concentration during exercise in the placebo trial (subgroup B) (Fig. 9B) did not terminate exercise prematurely in that (placebo) trial (Fig. 7D). A logical conclusion would be that CHO prevented the EIH causing fatigue in 7 of 10 placebo participants; yet, the authors concluded otherwise.

Ignoring the findings of the early Scandinavian researchers (see Figs. 4 and 5), Coyle et al (97) assumed that a decline in BG concentrations to 2.5 mmol/L, stage II hypoglycemia, was insufficient to impair performance in all participants since only 2 of the 7 individuals with this degree of EIH reported overt clinical symptoms of hypoglycemia. Since BG concentrations in the other 5 participants in subgroup A did not fall into these authors' self-defined “hypoglycemic range” of less than 2.5 mmol/L and since these individuals “complained primarily of severe weariness of the working muscles” during the control trial, the authors concluded that “CHO feeding may have delayed development of fatigue in these subjects by slowing muscle glycogen depletion.” However, emergent evidence exploring counterregulatory responses to hypoglycemia has demonstrated that hypoglycemic neurological and endocrine counterregulatory responses can occur at variable levels around and below 70 mg/dL (see evidence 6). This is critical, as the biological consequences of hypoglycemia are a direct reflection of the relative reduction in glucose near or below 70 mg/dL, but not universally attributed to a threshold of 70 mg/dL. It has been reported that even minor biological consequences, which may be unperceivable to the participants alongside the strain of exercise, can occur even with minor decreases in glucose that are not less than 70 mg/dL. They concluded that “fatigue was postponed by CHO feeding in 7 of the 10 subjects” but that “this effect appeared to be mediated by prevention of (*conventionally-diagnosed symptomatic*—*present authors' addition*) hypoglycemia in only two subjects.” Based on this interpretation, in a subsequent review (98) the authors concluded that during exercise of moderate intensity “when the reliance on carbohydrate for fuel is greater, carbohydrate feedings delay fatigue by apparently slowing the depletion of muscle glycogen” (p. 446). Without measuring muscle glycogen, the authors relied solely on participants’ complaints of muscle weariness without CHO ingestion. A more reasoned conclusion might have been that CHO ingestion improves the performance only of those whose BG concentrations fall progressively below the starting concentrations when they do not ingest CHO during exercise (compare lines A and B in Fig. 9A and 9B). This is the hypothesis that this review will interrogate.

Fig. 9 also shows that fatigue was associated with a progressive reduction in the %VO_2_max that the athletes could sustain even when fed CHO, which prevented the development of EIH. Thus, at the completion of both trials, exercise intensity had dropped to approximately 60% VO_2_max (Fig. 9D) or lower (Fig. 9C). Yet even Hultman and Bergstrom (5) believed that at these low exercise intensities, the oxidation of fat can provide all the energy required. Thus, the more logical explanation for these findings might be the following:

1. During prolonged exercise, the development of a falling BG concentration, below the concentration present at the start of exercise, produces a premature form of fatigue, the goal of which is to prevent glycopenic brain damage (28, 29).
2. This cannot be explained by an impaired skeletal muscle CHO energy metabolism since the associated reduction in exercise intensity below 60% VO_2_max, or even 50% VO_2_max (see Fig. 9C) should allow fat oxidation to substitute for CHO in providing any energy deficit.

Indeed, analysis of data from this specific study (97) (Table 3) confirms this speculative conclusion. These data show that at the point of fatigue when participants began to reduce their power outputs in both trials, the sole metabolic difference was the lower BG concentrations in individuals in the placebo trial.

**Table 3.** Rates of oxygen consumption (VO_2_), carbohydrates (CHO), and fat oxidation and blood glucose concentrations at exercise termination in 10 individuals when ingesting either placebo or CHO (140 g) during 3 hours of exercise at 74% maximum oxygen consumption (VO_2_max)

	Placebo control	CHO ingestion	Difference CHO ingestion – placebo control
VO_2_max (L/min)	^|^---- 4.0 ± 0.2 ----^|^		
Exercise VO_2_ (L/min)	2.96	2.96	0
Energy expenditure (kJ/min)	62.20	62.20	0
Respiration quotient (units)	0.89	0.89	0
CHO oxidation (g/min)	2.30	2.30	0
Fat oxidation (g/min)	0.60	0.60	0
Blood glucose concentration (mmol/L)	2.70	4.00	+1.3

**Figure 10.**
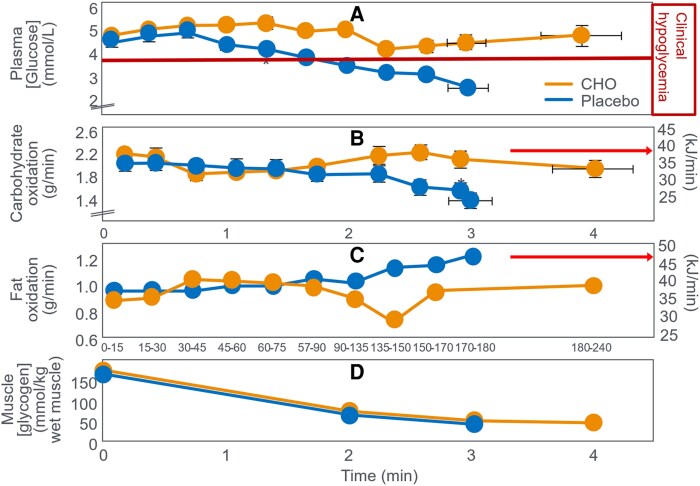
Changes in (A) plasma glucose, (B) rates of carbohydrate (CHO) and (C) fat oxidation, and (D) muscle glycogen concentrations, during prolonged exercise in athletes when they ingested either 100 g glucose/h or placebo during exercise. Data for fat oxidation rates were calculated from respiratory quotient (RQ) and oxygen consumption data published in the article using conventional equations. The energy derived from fat oxidation exceeded that from CHO oxidation for much of the experiment (arrows) even when CHO was ingested during exercise. Reproduced from (99).

These data establish that 140-g CHO ingested during 3 hours of exercise did not measurably alter participants' metabolism at the termination of exercise other than to maintain the BG concentration at or above the value at the start of exercise. The most logical explanation is that CHO ingestion prevented the development of the EIH, which impaired the exercise performance of 7 of the 10 individuals in the placebo trial. This interpretation aligns with the original interpretation of Boje, Christensen, and Hansen (27-29). Three years later (99), these same researchers included several important protocol changes. First, they selected a group of athletes with exceptional endurance, able to maintain a high-exercise intensity (71% VO_2_max) for at least 3 hours. The participants also began exercise after a 12-hour fast. Thus, while the hypothesis being tested was ostensibly the effects of CHO ingestion on exercise performance in athletes who began exercise with elevated muscle glycogen content because of eating a high-CHO diet, instead it evaluated the effects of CHO ingestion on exercise performance in those who began exercise with elevated muscle glycogen content but with reduced liver glycogen concentrations. Fig 10 shows the key findings of that study.

Fig. 10 shows that when they ingested the placebo, participants developed a progressive EIH and terminated exercise after 3 hours (Fig. 10A). CHO ingested at a rate of 100 g/h prevented EIH, extended the exercise duration by 1 hour, and produced slightly higher rates of whole-body CHO oxidation (Fig. 10B) with progressively lower rates of fat oxidation (Fig. 10C) but without differences in muscle glycogen use (Fig. 10D). This disproved the authors' prior claim that “CHO delays fatigue by slowing muscle glycogen depletion” (98).

Table 4, constructed from the data collected in that trial, shows that ingestion of 300-g CHO (5100 kJ) increased the average CHO oxidation rate by 0.20 g/min (3.4 kJ/min), increasing total CHO disappearance by 36.4 g (588 kJ) during the first 3 hours of exercise, accounting for 12% (619 kJ) of the total ingested CHO load. CHO ingestion reduced average fat oxidation rate by 0.10 g/min (3.8 kJ/min) for a total saving of 16.4 g (623 kJ) during 3 hours of exercise. After 3 hours of exercise, rates of CHO oxidation were 0.52 g/min (8.8 kJ/min) lower while fat oxidation rates were 0.23 g/min (8.7 kJ/min) higher with placebo compared to CHO ingestion. Table 4 also highlights the very high fat oxidation rates during exercise in these superior athletes (VO_2_max = 70 mL/kg/min) when they did not ingest CHO (Fig. 9C). This rate at 180 minutes (1.21 g/min) would have been among the highest reported in the scientific literature at that time although somewhat lower than values measured in Boje in 1939 (see Fig. 2D).

**Table 4.** Differences in total muscle glycogen disappearance and in total and average carbohydrate (CHO) and fat oxidation rates in individuals ingesting either placebo or 100 g CHO/h during 180 minutes of exercise at 71% maximum oxygen consumption (VO_2_max)

	Placebo ingestion	CHO ingestion	Difference (CHO vs placebo ingestion)
Total muscle glycogen disappearance in 180 min	129 glucose units/kg	126 glucose units/kg	−3 glucose units/kg
Total CHO (g) (kJ) (% total energy) oxidized during 180 min of exercise	326.5 g 5551 kJ 44%	361.1 g 6139 kJ 49%	+34.6 g + 588 kJ + 5%
Average rate of CHO oxidation (g/min) (kJ/min) during exercise	1.81 g/min 30.8 kJ/min	2.01 g/min 34.2 kJ/min	+0.20 g/min + 3.4 kJ/min
Rate of CHO oxidation (g/min) (kJ/min) at 180 min	1.38 g/min 23.5 kJ/min	1.90 g/min 32.3 kJ/min	+0.52 g/min + 8.8 kJ/min
Total fat (g) (kJ) (% total energy) oxidized during 180 min of exercise	181.9 g 6912 kJ 55%	165.5 g 6289 kJ 50%	−16.4 g −623 kJ −5%
Average rate of fat oxidation (g/min) (kJ/min) during exercise	1.01 g/min 38.4 kJ/min	0.91 g/min 34.6 kJ/min	−0.10 g/min −3.8 kJ/min
Rate of fat oxidation (g/min) (kJ/min) at 180 min	1.21 g/min 46.0 kJ/min	0.98 g/min 37.3 kJ/min	+0.23 g/min + 8.7 kJ/min

These data were calculated using the data in Fig. 9 (99).

Ingestion of 300-g CHO resulted in an essentially isocaloric substitution of CHO for fat oxidation, aligning with Fitts (34) that CHO ingestion during exercise produces a small effect on rates of CHO and fat metabolism and that “would not explain the complete exhaustion associated with prolonged exercise (*when CHO is not ingested*—*present authors' addition*).” They concluded that the 0.52 g/min (8.8 kJ/min) difference in the rate of CHO oxidation at 180 minutes represented “oxidizing CHO at relatively high rates from sources other than muscle glycogen.” This again raises the question: What unique role does this small additional amount of CHO oxidation play in sustaining performance once the exercising muscles are depleted of glycogen? And which, apparently, an equicaloric higher rate of fat oxidation (0.23 g/min; 8.7 kJ/min) could not—even though very high rates of fat oxidation had sustained exercise for the previous 3 hours in participants ingesting the placebo. During the total exercise bout, the 300 g of ingested CHO simply reduced total fat oxidation by 16.4 g (623 kJ) and increased CHO oxidation by an equicaloric amount of 34.6 g (588 kJ). There was a nearly exact energy swap from fat to CHO oxidation, though requiring ingestion of 300 g CHO (5100 kJ).

This analysis invites the recurring questions: How does the oxidation of an additional 36-g CHO during 3 hours of exercise, representing 952 kJ or just 8% of the total energy expenditure of 12 463 kJ, allow exercise to continue for another 60 minutes? How does the substitution of 8.8 kJ/min of CHO oxidation for 8.7 kJ/min of fat oxidation at the point of exhaustion prevent fatigue and allow exercise to continue for a further hour? Especially when this 8.8 kJ/min represents only 13% of the total rate of energy expenditure (69.6 kJ/min) at that moment. Exactly how does that “obligatory” extra 8.8 kJ from CHO oxidation sustain exercise since it is not “sparing” muscle glycogen use (Fig. 10D)? Why is a CHO oxidation rate of 1.90 g/min (32.3 kJ/min) able to sustain exercise for another 60 minutes whereas a rate of 1.38 g/min (23.5 kJ/min) causes incapacitating exhaustion and immediate exercise termination? The authors also calculated the energy contribution from muscle glycogen use during exercise (Table 5).

**Table 5.** Average energy contribution (kJ/min) from muscle glycogen use when carbohydrate or placebo was ingested during prolonged exercise

	Time, min									
	0		60		150		210		240	
	% energy	kJ/min	% energy	kJ/min	% energy	kJ/min	% energy	kJ/min	% energy	kJ/min
CHO ingestion during exercise	50	35	40	28	21	15	3	2	0	0
Placebo ingestion during exercise	57	40	40	28	18	13	11	8	0	0

Data extracted from Fig. 6 in (99).

Abbreviation: CHO, carbohydrate.

According to the theory that muscle glycogen provides the obligatory fuel during prolonged exercise, the conclusion from these data must be that fatigue occurred when between 3% and 11% of the required energy, representing 2 to 8 kJ/min, could no longer be provided by muscle glycogen metabolism. Yet at that same moment, CHO oxidation was providing 23 and 32 kJ/min and fat oxidation 46 and 37 kJ/min when placebo or CHO was ingested, respectively. The authors' complete conclusions were that: “… CHO feedings do not spare muscle glycogen utilization during intense continuous exercise” (p. 169); “lowering of blood glucose during the latter stages of prolonged strenuous exercise plays a major role in the development of fatigue by not allowing leg glucose uptake to increase sufficiently to offset reduced glycogen availability” (p. 170) so that “our present suggestion that hypoglycemia causes muscular fatigue when muscle glycogen is low is different from previous suggestions that hypoglycemia causes fatigue due to central nervous system dysfunction (28, 29)” (p. 170). As a result, “when they are fed CHO, highly trained endurance athletes are capable of oxidizing CHO at relatively high rates from sources other than muscle glycogen during the latter stages of prolonged strenuous exercise and that this postpones fatigue” (p. 165).

Thus: “Fatigue during prolonged (ie, ≥ 2 hours) exercise at 60% to 85% VO_2_max is often associated with an inadequate rate of CHO oxidation. By maintaining CHO oxidation during the later stages of exercise, the ingestion of CHO or other substrates before or during exercise has the potential to delay fatigue and enhance exercise performance (100 p.S334). This has since become the accepted explanation for the ergogenic effect of CHO ingestion during exercise. It is important to note the wide range of exercise intensities (60%-85% VO_2_max) to which this interpretation is applied. However, metabolism during prolonged exercise at 60% VO_2_max is quite different from that at 85% VO_2_max. While the claim that CHO is also the obligatory fuel for exercise at 85% VO_2_max might be reasonable, why should this apply, without justification, also to exercise at 60% VO_2_max since most accept that there is no metabolic limitation to the rate at which fat than can be oxidized at that exercise intensity (101)? Nevertheless, the authors concluded that “the primary mechanism by which CHO enhances endurance performance was due to a high rate of CHO delivery resulting in elevated rates of CHO oxidation” (102). In reality, however, “these relatively high rates” of CHO oxidation “from sources other than muscle glycogen” are remarkably small since they provided just 8.8 kJ/min comprising 8% of the total energy expenditure after 3 hours of exercise, during which time 5100 kJ (28 kJ/min) of CHO had been ingested. Furthermore, ingested CHO simply substituted for an identical amount of energy from fat oxidation in the placebo trial. Importantly, the authors acknowledge that their explanation was merely a “suggestion” (p. 170) because they provided no definitive experimental evidence to support this novel explanation that ignored the historical evidence (27-29). This interpretation ultimately replaced the longstanding explanation of Boje, Christensen, and Hansen (27-29).

**Figure 11.**
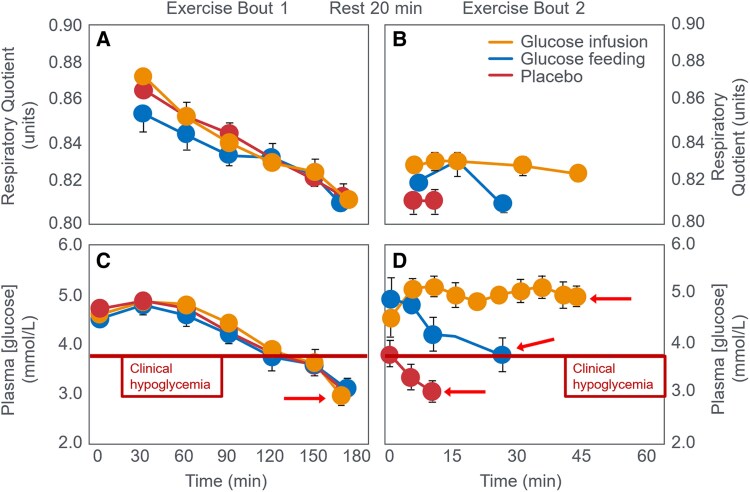
(A) Changes in respiratory quotient and (C) plasma glucose concentrations in individuals who initially exercised to exhaustion over 3 hours (exercise bout 1). Participants then rested for 20 minutes during which they received glucose or placebo by mouth or glucose by intravenous infusion. Thereafter they again exercise until exhaustion (exercise bout 2). (B and D) Show changes in respiratory quotient and plasma glucose concentrations during exercise bout 2. Reproduced from (103).

But this novel explanation appears incongruous with the finding that at exhaustion fat oxidation, likely increased by low muscle glycogen content (79, 80), was providing the majority of the energy in both interventions (Fig. 10C). Based on these data and the historical evidence (27-29), the more defendable hypothesis is that CHO acted by reversing or preventing the development of EIH (Fig. 10A). Instead, the authors' definitive conclusions helped establish the concept of the obligatory role of CHO oxidation during prolonged exercise even as the exercise intensity was falling to intensities at which fat oxidation would be able to provide most, if not all, the required energy. Despite exercising at 71% VO_2_max, highly fat-adapted athletes generated more energy from fat than CHO oxidation even after ingesting 6800 kJ CHO (Fig. 10B and 10C). In their next study (103), athletes exercised for 3 hours at 70% VO_2_max until clear EIH symptoms appeared. Participants then rested 20 minutes, receiving glucose orally, glucose intravenously (IV), or placebo before resuming exercise to exhaustion. CHO ingestion or infusion reversed EIH, allowing extended exercise based on the degree of EIH reversal (Fig. 11D).

The authors concluded that the “decline in plasma glucose contributes to fatigue during prolonged exercise *in part* (present authors' emphasis) by limiting CHO oxidation. This decline in CHO oxidation can be reversed and exercise continued for approximately 45 minutes, when euglycemia is restored and maintained by intravenous glucose infusion” (103 p.2388). This interpretation relies on the concept of “limiting CHO oxidation” as the sole metabolic factor restricting prolonged exercise performance. Table 6 lists the average rates of CHO and fat oxidation during exercise period 2, which followed the three different interventions.

**Table 6.** Metabolic responses to ingestion of glucose or a placebo, or to a glucose infusion at the point of fatigue during 3 hours of exercise at 70% maximum oxygen consumption

Rates of CHO and fat ox (g/min) (kJ/min)	Placebo		Glucose ingestion		Glucose infusion	
	CHO ox	Fat ox	CHO ox	Fat ox	CHO ox	Fat ox
After initial 180 min exercise	1.42 24.1	1.11 42.3	1.42 24.1	1.11 42.3	1.42 24.1	1.11 42.3
5 and 10 min after intervention	1.42 24.1	1.11 42.3	1.70 28.9	1.01 38.4	1.70 28.9	1.01 38.4
15 min after intervention	—		1.70 28.9	1.01 38.4	1.70 28.9	1.01 38.4
25 min after intervention	—		1.42 24.1	1.11 42.3	1.70 28.9	1.01 38.4
45 min after intervention	—		—		1.66 28.2	1.02 39.0

Abbreviations: CHO, carbohydrate; ox, oxidation.

CHO ingestion/infusion after 180 minutes raised CHO oxidation by 0.28 g/min (4.8 kJ/min), decreasing fat oxidation by 0.1 g/min (3.8 kJ/min). Once more this raises the persistent question: Exactly what obligatory role does this small contribution of increased CHO oxidation perform? And what is the evidence that this small difference is more important for exercise performance than is the reversal of EIH (Fig. 11D)? And why is it necessary that this energy must come from CHO rather than from fat oxidation which, it is now known, can support quite high rates of energy production even during high-intensity exercise (31, 101)? Additionally, what caused exercise termination 45 minutes after the start of the glucose infusion when metabolism had changed only imperceptibly (see Table 6) whereas the BG concentrations had normalized briefly (∼5 minutes) as a result of glucose ingestion or for approximately 45 minutes following glucose infusion (Fig. 11D)? However, the BG had risen little and fallen rapidly following placebo ingestion leading to fatigue and exercise termination within just 10 minutes (see Fig. 11D).

**Figure 12.**
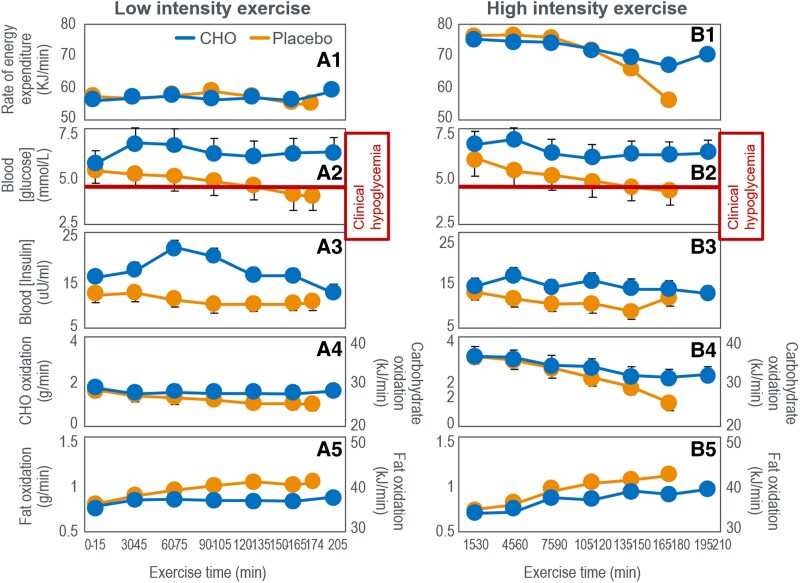
Panels on the left (A1-A5) are for exercise at 60% VO_2_max; those on the right (B1-B5) are for exercise at high intensity (85% VO_2_max). Carbohydrate (CHO) ingestion delayed the development of fatigue during high-intensity exercise (B1); prevented the development of a progressive EIH (A2 and B2); raised blood insulin concentrations (A3 and B3); and increased CHO oxidation (A4 and B4) while reducing fat oxidation (A5 and B5). Data for rates of fat oxidation were calculated from respiratory quotient and oxygen consumption (VO_2_) data in Coggan and Coyle (104) using conventional equations.

Coggan and Coyle (104) next studied trained cyclists performing alternating 15-minute intervals at 60% and 85% VO_2_max until exhaustion. In one trial, participants ingested a placebo; in the other, 64 g CHO after 10 minutes, then 40 g every 30 minutes. As a result, during 3 hours of exercise they ingested 300 g, the equivalent of 100 g/h, the same amount they had ingested in their previous study (99). Fig. 12A1 shows that participants maintained a constant energy expenditure (kJ/min) during the moderate-intensity exercise that lasted 174 minutes when placebo was ingested and 205 minutes with glucose ingestion. However, energy expenditure during the high-intensity intervals began to fall after 90 minutes both in the CHO and placebo trials (Fig. 12B1); the reduction was much greater for the placebo than for the glucose ingestion trial. CHO ingestion extended exercise duration by 31 minutes (18%).

**Figure 13.**
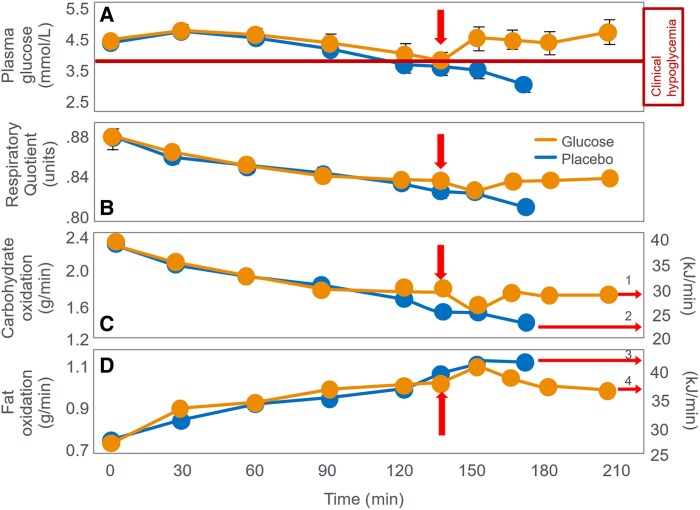
(A) Plasma glucose concentrations, (B) respiratory quotient, and (C) carbohydrate (CHO) and (D) fat oxidation rate during prolonged exercise when participants received either 210-g CHO or placebo at 135 minutes (vertical arrows). Data for CHO and fat oxidation were calculated from data in Table 1 in that paper (105) using conventional equations.

As expected, CHO ingestion (Fig. 12A and 12B) increased oxidation: +24 g during low-intensity and +36 g during high-intensity exercise (see Tables 1 and 2 (104)). This increase of 60 g (1020 kJ) represents approximately 15% of the total CHO (398 g; 6766 kJ) and 8% the total energy (13 382 kJ) used during exercise. It also represents just 20% of the total CHO energy ingested. CHO ingestion decreased fat oxidation by 12 g (low-intensity) and 11 g (high-intensity). Overall, ingesting 300 g CHO (5100 kJ) increased CHO oxidation by 60 g (1020 kJ) and reduced fat oxidation by 23 g (874 kJ). At the point of exercise termination at 174 minutes in the placebo group, athletes were oxidizing fat at 0.97 g/min (37 kJ/min) and CHO at 1.08 g/min (18 kJ/min) compared to values of 0.77 g/min (29 kJ/min) and 2.20 g/min (37 kJ/min) when CHO was ingested. The authors argue that it is this greater CHO oxidation (37 vs 18 kJ/min) that allowed participants to continue exercise for 33 minutes longer when they ingested CHO. Yet the key question remains: Why could not this extra 19 kJ/min be provided by fat oxidation (0.5 g/min)? The main effect of CHO ingestion was preventing progressive EIH after 90 minutes in the placebo trial (Fig. 12A2 and 12B2). When evidence of fatigue became apparent, at 120 to 135 minutes in the placebo group, the average BG concentrations had reached 3.8 mmol/L from a starting value of 5.4 mmol/L, falling further to 3.60 mmol/L at the termination of exercise. According to the studies represented in Figs. 4, 5, 6, 7, 8, 9, and 10, such low BG concentrations would be quite sufficient to explain the development of fatigue in the placebo group in this study. In their final cycling study, Coggan and Coyle (105) again had participants exercise at approximately 70% VO_2_max for 135 minutes after a 12- to 14-hour fast. Individuals then received either a placebo or 210-g CHO by mouth. Fig. 13A shows BG concentrations declined progressively after 60 minutes in both trials. CHO ingestion reversed EIH within 15 minutes, extending exercise duration by 36 minutes (21%) compared to placebo. These findings are essentially identical to those of Boje (27) (see Fig. 5) and Christensen and Hansen (29) (see Fig. 6).

These data show that CHO ingestion at 135 minutes maintained average CHO oxidation at 1.74 g/min to 1.73 g/min for the next 75 minutes (arrow 1); it did not increase that rate, in line with the findings of Boje (27) and Christensen and Hansen (29). CHO ingestion decreased fat oxidation from 1.01 g/min (40.9 kJ/min) to 0.97 g/min (37.0 kJ/min) at 210 minutes, a reduction of 0.4 g/min (15.2 kJ/min) (arrow 4) (Table 7).

**Table 7.** Metabolic responses to ingestion of 210 g of a glucose polymer or a placebo following 135 minutes' exercise at 70% maximum oxygen consumption

	210 g CHO ingestion at 135 min	Placebo ingestion at 135 minutes	Difference CHO vs placebo ingestion
Additional exercise duration (min) after 135 min	75	35	40
Total additional CHO oxidation (g) (kJ) during additional exercise time	130 2210	51 867	79 1343
Total additional fat oxidation (g) (kJ) during additional exercise time	73 2774	39 1482	34 1292
Rate of CHO oxidation (g/min) (kJ/min) at exercise termination	1.73 29.4	1.39 23.6	0.34 5.8
Rate of fat oxidation (g/min) (kJ/min) at exercise termination	0.97 36.9	1.11 42.2	0.14 5.3
Blood glucose concentration (mmol/L) at exhaustion	4.9	3.3	1.6

Abbreviation: CHO, carbohydrate.

Ingesting 210-g CHO at 135 minutes allowed participants to exercise 75 more minutes, oxidizing an extra 130 g CHO (2206 kJ) and 73 g fat (2774 kJ), whereas without CHO ingestion, CHO oxidation fell by 2.4 g/min (41 kJ/min) within 35 minutes while fat oxidation increased by 0.5 g/min (19 kJ/min) (Fig. 13C and 13D). As a result without CHO ingestion, participants exercised for only another 35 minutes during which time they burned an additional 51 g CHO (867 kJ) and 38.9 g fat (1476 kJ). At exercise termination, the respective rates of CHO oxidation were 1.39 g/min (24 kJ/min) without CHO ingestion and 1.73 g/min (29 kJ/min) with CHO ingestion. Rates of fat oxidation at this point were 1.11 g/min (42.1 kJ/min) without and 0.97 g/min (37 kJ/min) with CHO ingestion.

The question arises again: Why did CHO ingestion enable an additional 75 minutes of exercise? What obligatory role did the oxidation of that extra 0.34 g/min CHO (5.8 kJ/min) play when it represented less than 8.9% of the total energy expenditure at that time and especially when CHO was not the principal exercise fuel (compare arrows 4 and 1 and 3 and 2 in Fig. 13D)? Recall the claim that “the primary mechanism by which CHO enhances endurance performance was due to a high rate of CHO delivery resulting in elevated rates of CHO oxidation” (102). The authors concluded: “Interestingly, the extent to which fatigue was delayed in the present study (ie, by 30-60 minutes) by CHO ingestion late in exercise is like that observed in previous studies in which CHO was ingested throughout exercise. This suggests that **there may be no practical benefit to be gained by the ingestion of CHO supplements during exercise** (present authors' emphasis). Rather, what appears to be critical is the ability of such CHO supplements to supply glucose into the blood at sufficiently high rates late in exercise **to prevent a decline in their plasma glucose concentration and respiratory exchange ratio**” (present authors' emphasis) (105). They therefore reiterated their growing certainty: “These data indicate that a single CHO feeding late in exercise can supply sufficient CHO to restore euglycemia and increase CHO oxidation, thereby delaying fatigue (p. 59). In fact, neither RQ nor CHO oxidation rates increased after CHO ingestion (see Fig. 13B and 13C). Without this increase their explanation cannot be correct. They also did not explain why fat oxidation (35 kJ/min) remained the primary energy source after 60 minutes, even following CHO ingestion (arrows 3 and 4 vs 1 and 2 in Fig. 13C and 13D). The authors' interpretation contradicts their data and the original conclusions of Boje (27) and Christensen and Hansen (29) as the ingestion of CHO neither changed the RQ nor increased muscle CHO oxidation to any meaningful extent. Rather the rates of CHO oxidation were identical when measured at 135, 165, 180, and 210 minutes. Their assessment (103) showed a sustained increase in RQ following CHO provision at the point of exhaustion that required the glucose to be given via IV and for participants to rest for 20 minutes before they again resumed the exercise bout (Fig. 11B). The rest period and infusion vs oral ingestion likely altered CHO distribution.

### Summary

The reviewed experiments confirm Boje, Christensen, and Hansen's conclusions. CHO ingested during or at exhaustion extends exercise by preventing or reversing EIH, not by fueling obligatory CHO oxidation once glycogen is depleted. The evidence supporting this conclusion is the following:

- Fatigue during 2- to 4-hour exercise (65%-75% VO_2_max) usually coincides with falling BG in those fasting and not ingesting CHO.
- This fatigue typically occurs when fat oxidation, not CHO, dominates energy provision.
- This fatigue is alleviated by preventing or reversing EIH through CHO ingestion.
- CHO ingestion at exhaustion does not boost CHO oxidation significantly but reverses EIH, preventing further CHO oxidation decline (see Figs. 4, 5, 10, and 12).
- CHO ingestion primarily prevents EIH and results in an isocaloric substitution of CHO for fat oxidation (see Tables 3 and 4; Figs. 9 and 12).
- This explains Coggan and Coyle's finding that late CHO ingestion delayed fatigue similarly to continuous CHO ingestion (105). This effect arises from immediate replenishment of the bloodstream's SGP.
- This minor increase in whole-body CHO oxidation (see Tables 2-4, 6, and 7) does not fully explain how CHO ingestion significantly prolongs performance.
- As fat oxidation predominates even with CHO ingestion (see Figs. 2-6, 9-12), it is unclear how reducing fat oxidation enhances performance.
- Reversal of EIH requires a relatively small rate of CHO since the blood contains just 5 g of glucose (106, 107) (the SGP) and the rate of liver glucose appearance (Ra) from either ingested CHO or from liver glucose production needed to prevent EIH during prolonged exercise is probably less than 0.5 g/min (since brain glucose use at rest is ∼6 g/h [0.1 g/min] (108)).
- In fasted individuals, EIH produces premature fatigue before the onset of muscle glycogen depletion (109), the LGP. Currell et al (110) reported that participants who underwent a glycogen-depleting exercise trial lasting 2.5 hours followed by an overnight fast developed a progressive EIH that began within 15 minutes of starting exercise at 50% of peak work rate the following morning. This confirms glycogen-depleting exercise significantly reduces BG in subsequent fasted exercise.
- Importantly overlooked: fatigue in 1-hour high-intensity exercise is not linked to glycogen depletion or EIH (111).

The data from the series of experiments reviewed here confirms CHO ingestion during exercise (112, 113) is valuable by preventing BG decline, thus avoiding central nervous system reflex and glycopenic brain dysfunction, first proposed by Boje, Christensen, and Hansen in the 1930s. CHO ingestion benefits performance especially beyond 70 minutes (113), when EIH becomes increasingly probable (84, 98, 104, 105).

## Evidence 5: In Studies Showing CHO Improved Performance, BG Fell Progressively During Exercise in Up to 88% of Placebo Groups. Whereas in Studies in Which Exercise Performance Was Not Improved by CHO Ingestion, BG Concentrations Were Reduced in Only 30% of the Control Groups. CHO Ingestion During Exercise Improved Performance Approximately 2.7 Times More Often When BG Concentrations Fell in Control Groups

Fig. 9C (97) shows individuals prematurely fatigued when BG fell below initial levels (Fig. 9A).Thirty minutes later, participants reduced the exercise intensity they could sustain. This pattern was observed in multiple studies (104, 114-118). Conversely in that same original study (97), CHO ingestion did not improve performance in those whose BG concentrations remained at or above the starting value throughout the exercise bout whether or not they ingested CHO (Fig. 9B).

Emerging evidence suggests everyone has a threshold for hypoglycemia-related biological responses. In some individuals, these responses begin at BG concentrations greater than 4.0 mmol/L (Fig. 1 and 2 in (119)). Importantly, individuals can have no objective or subjective symptoms at levels at or slightly below 3.9 mmol/L, particularly in individuals who maintain chronically lowered albeit normal levels of glucose. However, it is established that individuals who maintain higher levels of glucose may experience hypoglycemia symptoms at levels greater than 3.9 mmol/L. We have previously demonstrated that dietary and metabolic factors contribute greatly to average glucose values, which may explain why habitual dietary patterns are strong determinants of EIH threshold (101). Nonetheless, symptoms are most reliable in individuals at or below 3.9 mmol/L, which is why medical definitions have consistently reported hypoglycemia below this threshold. Additionally, the magnitude of the neuroendocrine counterregulatory response appears to be dose dependent in relation to the relative BG drops, whereas lower glucose value elicit larger counterregulatory responses. Importantly, some of the counterregulatory changes can be subperceptive, particularly when elicited during strenuous exercise, often leaving athletes subjectively unaware of these biological consequences and their effects on performance, which may explain why some studies describe athletes “unaffected” by glucose values well below less than 70 mg/dL. The critical importance of normoglycemia is highlighted by minimal insulin strongly inhibiting hepatic glucose release.

The implications of this emergent evidence are as follows:

1. Hypoglycemic mg/dL or mmol/L thresholds for counterregulatory responses are different for each person and appear to be affected by the average glucose values of the individual.
2. Counterregulatory mechanisms can occur subperceptively, especially when compared to strenuous exercise, and thus may not be “subjectively detectable by participant,” despite their objective appearance in biological measurements and are likely contributing to reduced performance.
3. Relative drops in glucose from baseline or peak levels can biologically affect exercise metabolism and performance. Thus, the relative drop in glucose during exercise may have consequences that extend beyond an absolute threshold. Mitigations of glucose drops would then attenuate the manifestation of hypoglycemia, through the administration of continuous CHO sufficient to attenuate hypoglycemia from presenting, persisting, and progressing in magnitude, would have performance implications.

Consequently, this suggests that in terms of its effect on exercise performance, absolute BG, relative BG, and the change in glucose can have direct implications on exercise metabolism and performance that were unappreciated in prior analyses and that have important implications for the interpreted benefits of CHO during exercise that are independent of CHO oxidation and glycogen. EIH may be better defined as BG concentrations dropping below initial exercise levels.

To our best knowledge, there has not been an in-depth analysis of the frequency of either a falling BG concentration or frank EIH in the control and intervention groups in studies of the effect of CHO manipulations on exercise performance. The hypothesis presented in this review must predict that if a falling BG concentration contributes to impaired exercise performance, then it must occur more frequently in the control group in studies showing a beneficial effect of CHO manipulation on exercise performance.

For this analysis, we reviewed all the studies reported in Supplementary Tables S1 (Noakes, 2025 (120)) and S2 (Noakes, 2025 (120)) to determine the frequency with which BG concentrations fell during exercise in the control and intervention groups. We defined a falling BG concentration, indicating a developing EIH, as any reduction in the BG concentration greater than 0.1 mmol/L from the value measured at the start of exercise. This definition presumes that a central regulator of BG homeostasis during exercise (121) will respond to trends in BG concentration, especially the development of progressive EIH, by introducing protective responses to ensure that skeletal muscle uptake of BG is reduced well before a fatal neuroglycopenia can develop. The key response is to reduce the exercise intensity that can be sustained (Fig. 9C; Fig. 12B1), thereby reducing skeletal muscle glucose uptake. This anticipatory control contrasts with the catastrophic model of exercise regulation (60) in which such controls are galvanized only after the catastrophe—in this case neuroglycopenic brain damage—has already occurred. In Supplementary Tables S1 (Noakes, 2025 (120)) and S2 (Noakes, 2025 (120)), most studies in which we considered EIH to have developed, BG concentrations fell more than 0.3 mmol/L during exercise.

Of the 166 studies listed in Supplementary Table S1 (Noakes, 2025 (120)), BG concentrations were measured during exercise in 144 studies. In 97 studies (67%) (27-29, 72, 74, 84-86, 95, 97-99,103-105, 115, 116, 122-201)), BG concentrations fell in placebo groups during exercise. In another study (202), EIH could have been missed by delayed postexercise sampling. In 47 studies (33%), BG concentrations did not fall during exercise in the control group (73, 117, 202-246); in 22 studies BG concentrations were not measured (247-268) and in 1 (122) there was no control group. These findings are therefore compatible with the hypothesis that a falling BG concentration occurs frequently (67%) in studies of the effects of CHO manipulation on human exercise performance. Given the established consequences of a falling BG concentration on human exercise performance, it is surprising that this is not more widely recognized.

The performance effects of CHO ingestion were measured in 153 studies reported in Supplementary Table S1 (Noakes, 2025 (120)). Performance improved with CHO ingestion in 103 studies (67%) (27-29, 72, 74, 86, 97-99, 103-105, 115-117, 123, 124, 126-130, 133, 134, 137-139, 142-156, 158-160, 162-166, 172, 173, 175-186, 188, 189, 191, 193, 195, 198-202, 205-209, 212, 215, 220, 221, 223, 228, 232, 234, 235, 239, 242-244, 247, 249, 252, 256-260, 263, 264, 267), whereas in 50 studies (33%) (73, 85,95, 131, 132, 135, 136, 141, 157, 161, 170, 171, 174, 187, 190) (192, 194, 197, 201, 203, 210, 211, 213, 214, 216-218, 222, 224-227, 229-231, 237, 238, 240, 241, 245, 246, 248, 250, 251, 253-255, 261, 262, 266, 268, 269), performance was not altered by CHO ingestion. This confirms that CHO ingestion improved exercise performance in roughly two-thirds of reviewed studies.

In 72 of 103 studies (70%) showing CHO enhanced performance, BG concentrations fell in placebo groups (27-29, 72, 74, 86, 97-99, 103-105, 115, 116, 123, 124, 126-130, 133, 134, 137-139, 142-156, 158-160, 162-166, 172, 173, 175-186, 188, 189, 191, 193, 195, 198-201). This is compatible with the hypothesis that the prevention of a falling BG concentration in the control group is an important component of the beneficial effects of CHO ingestion on exercise performance. In contrast, among 40 studies with no CHO benefit, BG fell during exercise in only 17 (42%) placebo groups (85, 95, 115, 131, 132, 135, 136, 141, 157, 161, 170, 171, 174, 187, 190, 192, 194, 197) and remained in the normal range or elevated in 23 (58%) in the control group (203, 210, 211, 213, 214, 216, 217, 218, 222, 224, 225, 226, 227, 229, 230, 231, 237, 238, 240, 241, 245, 246, 270), perhaps indicating individual variability in susceptibility to the performance-impairing effect of developing EIH. Indeed, Lambert et al studied individuals adapted to a low-CHO diet continued exercising to lower BG (3.8 mmol/L) compared to higher CHO diets (4.2 mmol/L) (Fig. 2 in (271)).

BG concentrations were also measured in the (CHO ingesting) intervention group in 143 of the studies reported in Supplementary Table S1 (Noakes, 2025 (120)). During exercise, BG concentrations in the intervention group remained unchanged or increased in 120 (84%) of these studies (27-29, 72, 73, 84, 85, 95, 97-99, 104, 105, 115-117, 123, 124, 126, 128, 129, 131, 135-139, 142-152, 154-162, 165-173, 176, 178, 179, 181-186, 189-194, 196-199, 201-231, 233-246) and fell in 23 (16%) (74, 86, 103, 125, 127, 130-134, 140, 141, 153, 163, 164, 174, 175, 177, 180, 187, 188, 195, 200, 232). As expected, BG rarely fell (16%) in CHO-ingesting groups during exercise.

BG concentrations were also measured in the intervention group in 92 studies in which CHO ingestion improved the performance of the intervention group. In 75 (82%) of these studies, BG concentrations remained in the normal range during exercise (27-29, 72, 97-99, 104, 105, 115-117, 123, 124, 126, 128, 129, 137-139, 142-152, 154-156, 158-160, 162, 165, 166, 172, 173, 176, 178, 179, 181-186, 189, 191, 193, 198, 199, 201, 202, 205-209, 212, 215, 220, 221, 223, 228, 234, 235, 239, 242-244), whereas in 17 (18%) BG concentration fell during exercise in the intervention group (74, 86, 103, 127, 130, 133, 134, 153, 163, 164, 175, 177, 180, 188, 195, 200, 232).

BG concentrations were also measured in the intervention group in 38 studies in which CHO ingestion failed to influence performance. BG concentrations in the intervention group remained unchanged or increased in 36 (95%) of these studies (73, 85, 95, 131, 135, 136, 157, 161, 170, 171, 190, 192, 194, 197, 203, 210, 211, 213, 214, 216-218, 222, 224-227, 229-231, 237, 238, 240, 241, 245, 246) and fell in only 2 (5%) (141, 174, 187). These findings support the interpretation that a falling BG concentration in the intervention group reduces the likelihood that the study would produce a positive result of CHO ingestion on exercise performance.

Supplementary Table S2 (Noakes, 2025 (120)) lists studies of the effects on performance of CHO or glycerol or ketone ingestion either shortly before exercise or as part of a “CHO-loading” protocol. Of the studies included in Supplementary Table S2 (Noakes, 2025 (120)), the effect of the dietary intervention on exercise performance was measured in 124; in 74 studies (60%) (4, 31,48, 76, 114, 118, 139, 159, 205, 270-334) performance improved whereas in 50 studies (40%) (111, 269, 335-382) preexercise CHO supplementation did not influence exercise performance. In 3 studies (383-385) performance worsened, whereas in 13 studies (386-398) performance was not measured. These studies suggest preexercise CHO is less likely to improve performance than CHO ingestion during exercise. This supports the idea that altering the SGP is more effective for enhancing performance than manipulating the LGP before exercise.

This aligns with conserved glucose and nonglucose transport mechanisms in brain, liver, and muscle tissues, in which the primary glucose transporters in the brain are GLUT1 and GLUT3, and in the liver is GLUT2, all defined as passive transporters that allow for the transport of glucose into the cells of these tissues solely based on the concentration gradients across the extracellular and intracellular compartments and do not require energy input (ATP). Thus, brain and liver uptake glucose based on demand without energy input. However, skeletal muscle's primary GLUT transporter is GLUT4, which requires energy in the form of ATP (active transport) to move glucose into the skeletal muscle. Notably, insulin, a hormone present during nutritional abundance and that increases nutrient storage, is the primary mechanism by which skeletal muscle increases GLUT4 expression on the skeletal muscle membrane. Consequently, the body has conserved mechanisms to allow for preferential glucose uptake into critical tissue for mitigating hypoglycemia (ie, brain and liver) during high-energy demanding activities (exercise) or during reduced glucose availability (fasting or low-CHO) during which insulin is significantly reduced relative to normal levels. Conversely, muscle tissue requires energy (ATP) and energetic abundance (as signaled by insulin-induced GLUT4 translocation) to move glucose into the muscle providing conversed mechanisms to minimize GLUT uptake and thus utilization during environments where energy demands are increased (exercise), compromised (fasting), or altered (low-CHO). This is crucial since skeletal muscle, the body’s largest energy consumer, has substantially increased energy needs during exercise. This also aligns with evolutionarily conserved mechanisms for nonglucose substrates used by the brain, supporting its central role in regulating performance by conserving energy (see evidence 12). The presence of distinct passive vs active GLUT transporters across these 3 tissues also helps explain why CHO oxidation during exercise must progressively reduce during settings of high energetic demand (exercise) or low glucose availability (fasting and low-CHO), and is regulated by the starting levels of liver and muscle glycogen that is predominantly regulated prior periods of nutrient abundance and storage where insulin levels are elevated and energy demands are relatively reduced.

BG concentrations were measured in 101 of the studies and in 52 (51%) of these (4, 48, 114, 118, 139, 159, 271, 273, 283, 286-291, 293-296, 302, 305, 308, 309, 313, 317-323, 329, 331, 334, 339, 342, 351-353, 355, 358, 360, 366, 369, 384, 389, 391-395, 399) BG concentrations fell during exercise in the control group. In 49 (49%) studies, BG concentrations in the control group remained within the normal range during exercise (76, 270, 272, 274-276, 279-282, 284, 285, 299, 304, 310, 315, 316, 324, 325, 330, 335, 338, 340, 341, 343-347, 357, 359, 362-365, 367, 370, 375-378, 381-383, 386-388, 390, 392, 396). BG was not measured in the remaining studies (31, 111, 269, 278, 292, 297, 298, 300, 301, 303, 306, 307, 311, 312, 314, 326-328, 332, 333, 336, 337, 348-350, 354, 356, 361, 368, 371-374, 379, 380, 397, 398).

BG measurements were made in 56 of 75 studies in which performance improved with CHO supplementation. BG concentrations fell in the control group during exercise in 34 (61%) of these studies (4, 48, 114, 118,139, 159, 271, 273, 283, 286-291, 293-296, 302, 305, 308, 309, 313, 317-323, 329, 331, 399), with normal BG concentrations in 22 (39%) studies (76, 270, 272, 274-277, 279-282, 284, 285, 299, 304, 310, 315, 316, 324, 325, 330, 334). This is in keeping with the previous finding that exercise performance is more likely to be improved by CHO manipulation if the BG concentration falls during exercise in the control group.

BG concentrations were measured in 34 of 50 studies in which performance was not improved by CHO ingestion. BG concentrations fell in the control group in 11 studies (32%) (339, 342, 351-353, 355, 358, 360, 366, 369, 399), whereas BG concentrations remained normal or elevated in the control group in 23 (68%) studies (335, 338, 340, 341, 343-347, 357, 359, 362-365, 367, 370, 375-378, 381, 382). This too supports the interpretation that exercise performance is less likely to be improved by CHO ingestion if BG does not fall during exercise in the control group.

BG concentrations were measured in the (CHO ingesting) intervention group in 102 of the studies reported in Supplementary Table S2 (Noakes, 2025 (120)). During exercise, BG remained unchanged or increased in the intervention group in 60 (59%) of these studies (76, 114, 159, 270, 272-277, 279, 281-285, 294, 299, 304, 308-310, 315-317, 319, 321-325, 334, 335, 338, 340, 341, 344-346, 353, 357, 359, 360, 362, 363, 365, 367, 375-378, 381-383, 386-388, 390, 391, 396, 399) and fell in 42 (41%) (4, 48, 118, 139, 271, 280, 286-291, 293, 295, 296, 302, 305, 313, 318, 320, 322, 329-331, 339, 342, 343, 347, 351, 352, 355, 358, 364, 366, 369, 370, 384, 385, 389, 392, 394, 395).

BG concentrations were measured in the intervention group in 34 studies in which CHO ingestion failed to influence performance. BG concentrations remained unchanged or increased in the intervention group in 22 studies (65%) (335, 338, 340, 341, 344-346, 353, 357, 359, 360, 362, 363, 365, 367, 375-378, 381-383), and fell in 12 studies (35%) (339, 342, 343, 347, 351, 352, 355, 358, 364, 366, 369, 370).

BG concentrations were measured in the intervention group in 54 studies in which performance was improved. In 31 (57%) of these studies, BG concentrations in the intervention group remained in the normal range during exercise (76, 114, 159, 270, 272-277, 279, 281-285, 294, 299, 304, 308-310, 315-317, 319, 321, 323-325, 399); whereas in 23 (43%) BG concentrations fell during exercise (48, 118, 139, 271, 280, 286-291, 293, 295, 296, 302, 305, 313, 318, 320, 322, 329-331).

These studies suggest that, compared to a clear effect on exercise performance in the control group, any reduction in BG concentrations in the intervention group has a much smaller or perhaps negligible effect on whether the CHO intervention will produce a measurable effect in enhancing exercise performance (in the control group). This supports the hypothesis that CHO's influence on exercise performance depends on preserving BG concentrations during exercise in the control group. Whether CHO prevents a fall in the BG concentration during exercise in the control group, this effect arises solely by preventing depletion of the SGP.

### Summary

Table 8 provides a combined analysis of the effects of CHO ingestion and exercise on BG concentrations in the control and intervention groups in 217 studies from Supplementary Tables S1 (Noakes, 2025 (120)) and S2 (Noakes, 2025 (120)) that either produced or failed to result in an improved exercise performance. The goal of the analysis is to establish the frequency with which a falling BG concentration develops in the control or intervention groups or both in studies of the effects of CHO manipulations on exercise performance.

**Table 8.** Changes in performance as related to changes in blood glucose concentrations in both the intervention and control groups in studies of carbohydrate manipulation before (Supplementary Table S2 [Noakes, 2025 (120)] or during exercise (Supplementary Table S1 [Noakes, 2025 (120)]) across 167 studies

		Performance improved by CHO ingestion (studies)	Performance unchanged by CHO ingestion (studies)
A	BG concentrations reduced in control group but stable in intervention group	63 (29%) Table 1: 52 Table 2: 11	7 (3%) Table 1: 6 Table 2: 1
B	BG reduced in both control and intervention groups	42 (19%) Table 1: 20 Table 2: 22	13 (6%) Table 1: 5 Table 2: 8
C	BG concentrations stable in control and intervention groups	40 (18%) Table 1: 19 Table 2: 21	47 (22%) Table 1: 29 Table 2: 18
D	BG stable in control but decreased in intervention group	2 (1%) Table 1: 0 Table 2: 2	3 (2%) Table 1: 0 Table 2: 3

Abbreviations: BG, blood glucose; CHO, carbohydrate.

Table 8 addresses the hypothesis that CHO ingestion is more likely to influence exercise performance if the BG concentration falls in the control/placebo group during exercise. This supports the idea that fatigue during prolonged exercise is primarily driven by depletion of the SGP. This analysis shows that in 105 of 125 studies (88%) (Table 8A and 8B) in which BG concentrations were reduced in the control group, CHO ingestion improved performance. In contrast, if the BG concentration was stable in the control group, there was an essentially equal probability that the study would be associated with either an improved (18%) or an unchanged (22%) exercise performance (Table 8C). These findings support the hypothesis that a major determinant of the beneficial effect of CHO ingestion on exercise performance is the prevention of a falling BG concentration during exercise. Surprisingly, these findings are rarely discussed despite Boje, Christensen, and Hansen establishing in 1939 (27-29) that preventing or reversing BG decline delays fatigue during exercise.

#### Meta-analysis of the nature of carbohydrate composition of the preexercise meal

A recent meta-analysis (400) found a preexercise meal glycemic index significantly influenced exercise performance, independent of CHO ingestion during exercise (401). However, a small, nonsignificant performance benefit was observed after a low-glycemic index meal when no CHO was ingested during exercise. This is compatible with the interpretation that the prevention of EIH is the main ergogenic benefit of CHO ingestion either before or during exercise, since the sole effect of the preexercise low-glycemic index meal would be to increase the size of the SGP.

#### Effect of preexercise carbohydrate-loading

Concerning preexercise CHO-loading, 19 studies (48, 76, 114, 118, 273, 275, 299-304, 314-317, 329, 332-334) reported a positive effect on performance while 16 (111, 269) (348, 354-358, 371, 378-382, 384, 399) found no beneficial effect of CHO-loading on performance. A further 7 studies (31, 359, 360, 361, 367, 368, 374) that compared the effects of habitually high or low CHO diets found no such effect. In 2 studies there was no performance measurement (397, 398). Jensen et al (48), replicating the iconic 1967 study (4), found profound EIH developed in individuals on the lowest CHO diet. Yet at exhaustion, participants with EIH were still oxidizing CHO at a high rate (2.6 g/min) (relevant entry in Supplementary Table S2 (Noakes, 2025 (120)) despite marked hypoglycemia, a finding that is not predicted by the novel obligatory CHO replacement hypothesis. Erlenbusch et al (400) noted trial variability means “a decisive endorsement for a high-CHO diet can't be confidently offered, especially for trained athletes” (p. 31). Sherman and Wimer (401) had reached a similar conclusion 14 years earlier but nevertheless felt it “prudent to advise athletes to consume a diet with a high carbohydrate content” since there was no evidence that a high-CHO diet impairs performance (p. 28). In other words, lack of evidence for CHO-loading harm is often accepted as proof of benefit.

This lack of definitive evidence linking higher preexercise glycogen to superior performance challenges the idea that muscle glycogen is an obligatory fuel, as commonly concluded from Fig. 1. Interestingly, studies show increased training loads reducing glycogen do not consistently impair training performance (355, 401-404).

## Evidence 6: EIH Affects Performance Via an Integrated Mechanism Involving Hypothalamic Pathways Linking Blood Glucose to Skeletal Muscle Motor Unit Recruitment (SMMUR) When BG Falls Below Resting Levels. Controlling SMMUR in This Way Protects Against a Fatal Neuroglycopenia by Reducing the Rate of BG Disappearance (Rd) When Rates of Glucose Appearance (Ra) Become Limiting

**Figure 14.**
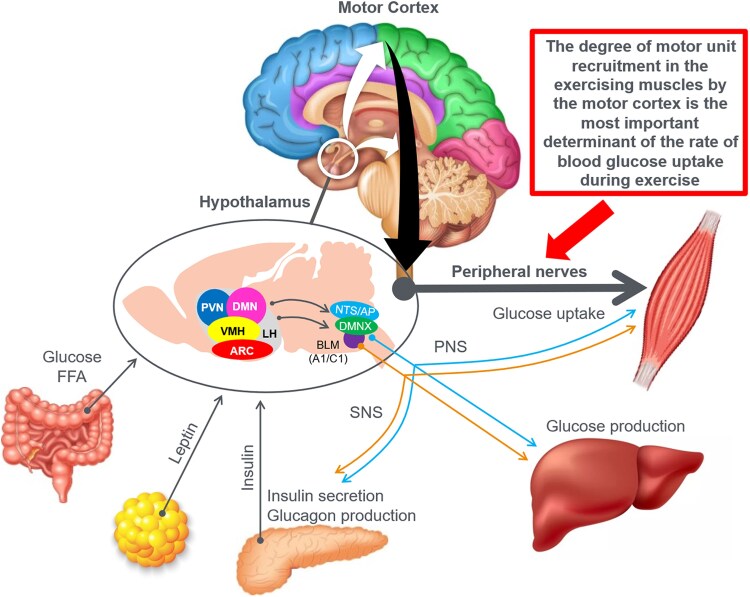
Redrawn from (121) with additions based on reference (408). Specialized brain areas in the hypothalamus and brainstem (AP, area postrema; ARC, arcuate nucleus; BLM, basolateral medulla; DMN, dorsomedial nucleus; DMNX, dorsal motor nucleus of the vagus; LH, lateral hypothalamus; NTS, nucleus of the solitary tract; PNS, parasympathetic nervous system; PVN, paraventricular nucleus; SNS, sympathetic nervous system; VMH, ventromedial hypothalamus) sense peripheral metabolic signals through hormones and nutrients to regulate glucose metabolism. The autonomic nervous system contributes by modulating pancreatic insulin/glucagon secretion, hepatic glucose production, and skeletal muscle glucose uptake. Text copied from reference (121). Missing from this diagram is a necessary link between these hypothalamic, brainstem, and motor cortical structures specifically to prevent excessive skeletal muscle motor unit recruitment during prolonged exercise when liver glycogen depletion develops, causing progressive exercise-induced hypoglycemia (EIH). Without this added control, all forms of prolonged exercise would ultimately cause EIH (95, 106, 107, 409) and hypoglycemic brain damage (407). Also missing is the effect of the blood glucose concentration in directly regulating hepatic glucose production (410, 411).

Cahill (405) argues there are “several general metabolic guidelines relating to fuel homeostasis….One of these is to maintain (blood) glucose levels within very narrow limits, returning the level rapidly to the norm if perturbed in either direction” (p. 785). The Central Governor Model of Exercise (60, 406) explains why exercise performance is always regulated “in anticipation” by a brain mechanism that protects the body from harm. Hypoglycemic brain damage is one such major threat (407), perhaps the greatest such threat during very prolonged exercise if CHO is not ingested. The model describing brain (hypothalamic) mechanisms for the homeostatic regulation of blood glucose concentrations (121) under resting condition, does not include a mechanism to regulate SMMUR, which is the major determinant of blood glucose Rd during exercise. This mechanism limits SMMUR and BG oxidation as EIH develops when liver and intestinal rates of Ra are falling. Thus, SMMUR must be integral to mechanisms maintaining BG homeostasis during exercise. Fig. 14 depicts a likely model.

When hypothalamic areas detect falling BG, central motor drive to muscles reduces to avoid hypoglycemic brain damage (407). This explains why performance declines progressively as BG inevitably falls during prolonged exercise (4, 29, 72, 73, 85, 86, 97, 99, 103, 105, 114-117, 126-130, 133-135, 137-139, 147, 149, 151, 154, 155, 158, 159, 163, 172, 175, 176, 178, 179, 181, 182, 185, 186, 188, 189, 193, 195, 196, 200, 204, 209, 237, 260, 265) in those who do not ingest CHO during exercise (57). The studies by Nybo and colleagues (412-414) clearly establish that EIH impairs cerebral glucose and oxygen uptake and consequently impairs the ability of the motor cortex, hypothalamic, and midbrain structures to activate skeletal muscle, exactly as predicted by this model. Conversely, the studies by Newell et al (182, 186) (see also Table 8 subsequently) found that the ergogenic effects of low doses of CHO were as effective as larger doses; they subsequently could not find evidence that, when tested in recovery after exercise, higher CHO intake during exercise produced different neural control responses than did lower CHO intake (415). Since postexercise testing using short duration, maximal contractions of the quadriceps muscles and does not threaten BG regulation, it would be predicted that alterations in neural responses directed to protect BG regulation during prolonged exercise would not be apparent when those controls were no longer necessary.

## Evidence 7: Recommended CHO Ingestion Rates for Athletes Have Risen Dramatically Without Clear Biological Evidence of Their Benefits. Nor is There Evidence Athletes Routinely Adopt These High Ingestion Rates

Before the first studies of the effects of the coingestion of multiple separate CHO sources on endogenous CHO oxidation rates, the general opinion was that CHO rates of intake greater than 16 g/h did not produce greater benefits than lower values. Thus: “ingesting CHO at a rate higher than 75 g/hr did not appear to be any more effective at improving performance than ingesting CHO at a rate of 40 to 75 g/hr … The overall conclusion seems to be that performance benefits can be observed with relative (sic) small amounts of CHO (16 g/hr), but no further improvements have been observed with the ingestion of larger amounts of CHO” (416 p.670). “To our knowledge, no studies have demonstrated that ingesting larger amounts of CHO that will result in higher exogenous CHO oxidation rates will also enhance performance. Studies have shown that effects of CHO feeding even with relatively low rates of intake (as low as 16 g/h), but generally no greater improvements have been observed with higher intake rates” (416 p.675). This opinion conflicted with the semi-official 1992 drinking guidelines that promoted rates of CHO ingestion of 64 to 128 g/h during more prolonged exercise (417). Nor did it support the novel 1986 replacement hypothesis that the beneficial effect of CHO ingestion during exercise is the result of “relatively high rates of CHO oxidation from sources other than muscle glycogen” (99 p.165). Alternatively, low rates of CHO intake (16 g/h) might be sufficient to prevent EIH since the blood contains so little (5 g) glucose (106, 107) and the rate of turnover of this GP is substantially slower than the rate of turnover of much large muscle glycogen/GP (see later).

In 2008, Jeukendrup (418) proposed an increase in the lower limit of the rate of CHO intake to improve performance, to 60 to 70 g/h. Four years later he revised this to 90 g/h (419), the same rate promoted in a coauthored paper (420) as well as by Cermak and Van Loon (421). In 2014, Jeukendrup (422) increased his range of optimum CHO intake rates to 60 to 90 g/h and in the same year Stellingwerff and Cox (102) suggested increasing this rate further to more than 90 g/h. Five years later, Burke et al (423) also suggested optimum rates of 75 to 90 g/h whereas Costa et al (424) advised ultramarathon runners to ingest CHO at a rate of 90 g/h. In 2021, Bourdas et al (425) concluded that the optimal CHO dose is 80 to 100 g/h, while the most recent (2022) review advises intake rates of 120 g/h (426, 427). More recently, Jeukendrup (428) has suggested that the optimal rate of CHO ingestion during exercise is determined by the duration of the exercise, increasingly linearly from 60 to 120 g/h as the exercise duration increases from 1 to 3 hours. A recent modeling exercise by Lukesiewicz et al (429) concludes that: “Exogenous CHO intakes ≤than 90 g/h are insufficient for 65% of modelled runners attempting (to run) a S2M (sub-2hr marathon).” It is difficult to reconcile this advice with the 1989 conclusion that high rates of CHO ingestion during exercise “may be of no practical value” (105) or indeed with the real-life practices of athletes in actual competitions. For example, studies of ultraendurance athletes following ad libitum fluid and energy replacement seldom report CHO intake rates greater than 30 to 60 g/h (202, 430-452), with most rates at the lower end of the range; the highest CHO intake rates in Ironman triathletes seldom exceeded 60 g/h (see Tables 1 and 3 in (453); Table 3 in (454)) although somewhat higher rates of up to 70 g/h were measured in the New Zealand Ironman Triathlon (455). Other studies match these findings. An earlier (1998) study of cyclists in the Tour of Spain reported CHO intake rates of just 25 g/h and fat intakes of just 4 g/h (450). Reports of individuals ingesting higher rates of CHO during ultramarathon running especially (456, 457) appear to be outliers or were achieved by athletes participating in experimental trials where 120 g/h was dictated by the experimental protocols (262, 263).

This exclusive focus on CHO ingestion during exercise ignores the finding that finishers in the Western States 100-mile Mountain Footrace have higher rates of total energy, CHO, and fat ingestion; including up to 50 g (1873 kJ) more fat during the race than do nonfinishers (432). Rates of CHO intake in finishers and nonfinishers in this race were respectively 66 and 42 g/h (432), whereas rates of fat intake were nearly 3 times higher in finishers (3.6 g/h) than in nonfinishers (1.3 g/h). Interestingly, rates of CHO intake of 45 g/h appear to be well tolerated but “higher rates (up to 90 g/h) of multiple-transportable carbohydrate intake during running appear to be less tolerable (458) despite their recommended intake (459)” (460). In general race diets that include more CHO (461, 462) and less fat (432, 453, 463) are more likely to be associated with gastrointestinal distress during competition. Indeed the most common explanation by athletes unable to ingest CHO at 90 g/h during ultramarathon races is because of “the persistence of gastrointestinal symptoms and/or appetite suppression” (454).

### Summary

In summary, recommended CHO ingestion during exercise rose dramatically over 2 decades from 16 to 120 g/h. This increase lacks definitive biological evidence that high CHO rates enhance ultraendurance performance. Rather it is the prediction made from the novel replacement model that postulates that once muscle glycogen depletion occurs, the exercise can continue only if exogenous CHO is provided at high rates. Yet even Coggan and Coyle noted that “plasma glucose becomes the predominant CHO energy source in the latter stages of prolonged exercise at ∼70% VO_2_max” (103) at the precise time when “the rate of (glucose) entry into the blood was unable to match its rate of removal” (103). This is the perfect physiological recipe for the development of EIH (82, 83, 176, 409), which, this review argues, is the most important biological mechanism by which CHO-depletion impairs human exercise performance. Also ignored by these recommendations is the evidence that such high rates of CHO ingestion produce, in otherwise healthy athletes, a state analogous to that found in obese, prediabetic individuals with marked insulin resistance shown by elevated blood insulin concentrations, unphysiologically low rates of fat oxidation, and a marked dependence on muscle glycogenolysis for energy production (464) (See Figs. 19 and 21 subsequently).

## Evidence 8: Increasing CHO Ingestion Boosts CHO Oxidation Rates, But No Clear Dose-response Effect on Performance Exists

The hypothesis by Coyle et al (39, 40, 100) that CHO ingestion delays or reverses fatigue during prolonged exercise by providing an obligatory source of CHO when muscle glycogen stores are depleted (34, 39, 40, 425) must predict that the highest rates of exogenous CHO oxidation will produce the greatest ergogenic benefit: “the lowering of blood glucose during the latter stages of prolonged strenuous exercise plays a major role in the development of muscular fatigue **by not allowing leg glucose uptake to increase sufficiently to offset reduced muscle glycogen availability** (present authors' emphasis) (99, 420)”. So that: “If carbohydrate ingestion is to improve endurance performance, likely, the beneficial effect is primarily dependant on the oxidation of that carbohydrate” (418 p.79). Thus: “The amount of CHO that needs to be ingested to obtain optimal performance is important from a practical point of view. The optimal amount is likely to be the amount of CHO resulting in maximal exogenous CHO oxidation rates” (465 p.415) since “A greater contribution of exogenous (external) fuel sources (CHO) will spare endogenous sources, liver and possibly muscle glycogen in some conditions, and it is tempting to believe that a greater contribution from exogenous sources will increase endurance capacity and/or exercise performance. Although many studies (including our own) are based on this assumption, **the evidence for this is lacking** (present authors' emphasis)” (416 p.675). And: “… fatigue during prolonged exercise is often due to inadequate carbohydrate oxidation. This is sometimes a result of hypoglycemia which limits carbohydrate oxidation and causes muscle fatigue” (466 p.S128). Furthermore, this maximal rate is in turn ultimately “limited” by the rate of intestinal CHO absorption (465, 467, 468) since “studies indicate the importance of intestinal absorption of CHO as a primary ***limiter*** (present authors' emphasis) of ergogenic effects” (468).

**Figure 15.**
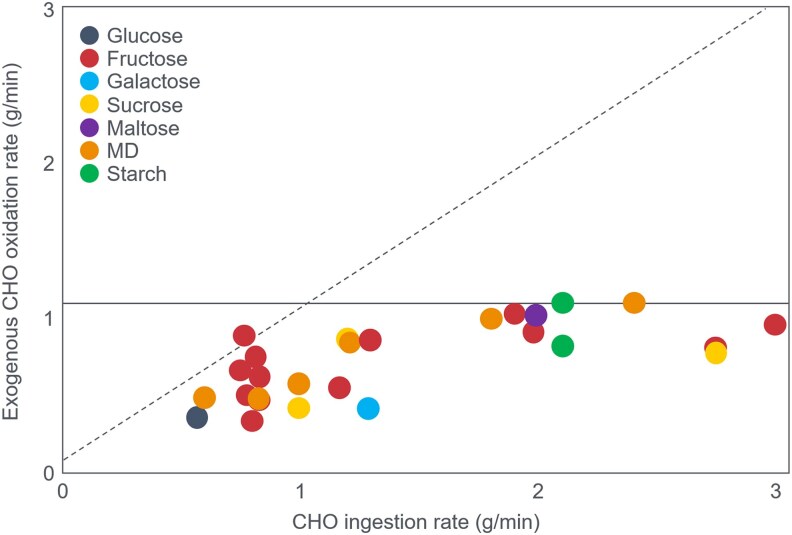
Exogenous carbohydrate (CHO) oxidation rate increases as a function of the CHO ingestion rate up to an apparent maximum at an ingestion rate of 1 g/min. When ingested as single CHO sources, there is no difference in endogenous CHO oxidation rates from glucose, fructose, galactose, sucrose, maltose, maltodextrin, or starch. Reproduced from (465).

These models predict that after muscle glycogen depletion, CHO use primarily comes from exogenous sources via the bloodstream, especially after liver glycogen depletion. If correct, this predicts that as the rate of CHO ingestion increases during exercise, the rate of exogenous CHO oxidation and the subsequent improvement in exercise performance will increase in parallel. This prediction led to many studies assessing how different CHO solutions influence exogenous CHO oxidation rates across various exercise intensities and durations (Supplementary Table S3 [Noakes, 2025 (120)]) (79, 80, 100, 125, 165-167, 172, 176, 178, 179, 185, 189, 194, 204, 207, 210, 219, 249, 254, 258, 259, 319, 335, 399, 423, 425, 429-466, 469-472). Early studies (465) found an apparent limit to the rate of exogenous CHO oxidation when single-CHO sources (eg, glucose, fructose, sucrose, maltose, maltodextrin) are ingested (Fig. 15). This approximately 1-g/min limit occurred when the ingestion rate was also approximately 1 g/min. Wagenmakers et al (473) found that increasing the rate of maltodextrin ingestion from 0.6 to 2.4 g/min increased rates of oxidation from 0.53 to 1.07 g/min with a leveling off above an intake of 1.2 g/min. Whereas 72% of the CHO ingested at 0.6 g/min was oxidized, only 32% of the highest CHO load (2.4 g/min) was similarly oxidized. In retrospect, this finding is not compatible with the hypothesis that, when glycogen-depleted, exercising skeletal muscle must maximize its extraction of exogenous CHO.

Subsequently, Jeukendrup et al (474) measured rates of exogenous CHO oxidation in individuals ingesting glucose at rates of 1.2 to 3.0 g/h. Rates of exogenous CHO oxidation rose from 0.34 to 0.94 g/min with an increasing dose of ingested CHO. Yet, the oxidized percentage of ingested CHO dropped from 59% to 21%. The authors reported the highest glucose intake completely suppressed liver glucose production. Total CHO oxidation rose with more ingested CHO, while fat oxidation progressively declined. They concluded that “the entrance of glucose into the systemic circulation (*presumably from the gut*—*present authors' addition*) seems to be the limiting factor for exogenous CHO oxidation …”. This suggests intestinal control may prevent rapid entry of ingested glucose into the systemic circulation during exercise. Such a control would act to prevent catastrophic hyperglycemia with very high rates of exogenous CHO ingestion, but which did not occur in that study.

While the study acknowledged that high rates of CHO ingestion reduced the rates of fat oxidation compared to exercise without CHO ingestion, the relentless search for the very highest rates of exogenous CHO oxidation shifted the focus exclusively to interventions that would maximize CHO oxidation during exercise. Clear evidence showing fat oxidation supports prolonged exercise (see Figs. 2 and 9; see also evidence 15 and 16) and CHO ingestion inhibits fat oxidation was largely overlooked. Yet when properly analyzed, numerous studies had already established that rates of fat oxidation at fatigue exceeded rates of CHO oxidation (eg, Tables 1, 2, 4, 6, and 7; Figs. 2, 4, 5, 9, 11, and 12). Since simply increasing the amount of CHO ingested could not increase the rate of exogenous CHO ingestion to more than 1.0 g/min (Fig. 15), the next development was to study the effects of the coingestion of multiple CHO sources. Thus, early studies found that the addition of fructose (475) or sucrose (476) increased peak oxidation rates to approximately 1.3 g/min, perhaps because fructose and (undigested) sucrose are absorbed by intestinal transport systems different from the glucose GLUT5 transporter.

In the next advance, Jentjens et al (475) reported that coingestion of glucose, fructose, and sucrose at 2.4 g/min (144 g/h) increased the exogenous CHO oxidation rate to approximately 1.7 g/min. Lower rates were measured with coingestion of glucose and sucrose (1.3 g/min) or glucose and maltose (1.1 g/min) (476) or glucose and fructose (1.3 g/min) (475). But what these authors may have missed was that CHO ingestion caused an essentially isocaloric substitution kJ for kJ of the increased CHO oxidation with a reduced fat oxidation.

**Figure 16.**
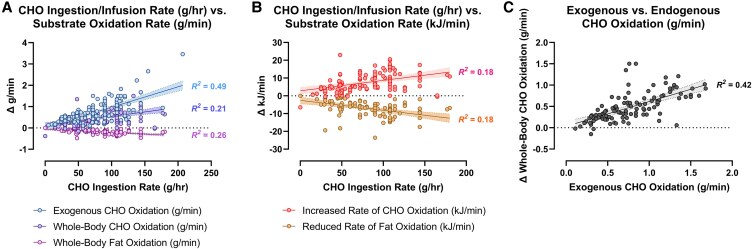
A shows the effect of increasing total carbohydrate (CHO) ingestion on rates of exogenous CHO, whole-body CHO, and fat oxidation reported in g/h. B shows the effect of increasing total CHO ingestion on rates of CHO and fat oxidation reported in kJ/min. C shows the effects of increasing exogenous CHO oxidation (g/min) on changes in whole-body CHO oxidation (g/min). These are based on an analysis of data in Supplementary Tables S3 (Noakes, 2025 (120)) and S4 (Noakes, 2025 (120)). The data were analyzed using a commercially available statistical package (GraphPad PRISM, ver. 9.3.0) to explore the relationship between exogenous CHO ingestion rate and changes in endogenous CHO/fat oxidation with a series of simple linear regression tests. Each regression model included approximately 154 cases; replicate values or missing cases were handled automatically by the statistical software (94% valid cases). The goodness of fit of each model is reported as R-squared. The clouded area around the regression line denotes the 95% CI.

Supplementary Tables S3 (Weltan, 1998 (79); Weltan, 1998 (80); Jeukendrup, 2006 (165); Jentjens, 2006 (167); Wallis, 2007 (168); Currell, 2008 (172); Smith, 2010 (176) Newell, 2015 (182); King, 2018 (185); Newell, 2018 (186); King, 2019 (189); Rowe, 2022 (195); Podlogar, 2022 (196); Rowlands, 2008 (227)); Cox, 2010 (231); Triplett, 2010 (244); Hearris, 2022 (264); Margolis, 2019 (397); Hawley, 1994 (464); Wagenmakers, 1993 (473); Jeukendrup, 1999 (474); Jentjens, 2004 (475); Jentjens, 2004 (476); Bosch, 1994 (477); O’Hara, 2012 (478)); Trommelen, 2017 (479); Hawley, 1994 (480); Jeukendrup, 1999 (481); MacLaren, 1999 (482); Wallis, 2006 (483); Leijssen, 1995 (484); Derman, 1996 (485); Coyle, 1991 (486); Bosch, 1996 (487); Adopo, 1994 (488); Burelle, 2006 (489); Couture, 2002 (490); Hawley, 1992 (491); Hulston, 2009 (492); Jandrain, 1984 (493); Jandrain, 1989 (494); Jentjens, 2005 (495); Jentjens, 2004 (496); Jentjens, 2005 (497); Jentjens, 2002 (498); Krzentowski, 1984 (499); Krzentowski, 1984 (500); Massicotte, 1994 (501); Massicotte, 1986 (502); Massicotte, 1989 (503); Massicotte, 1992 (504); Mohebbi, 2020 (505); Moodley, 1992 (506); Mosora, 1981 (507); Odell, 2022 (508); Odell, 2020 (509); Pallikarakis, 1986 (510); Pettersson, 2020 (511); Pirnay, 1982 (512); Pirnay, 1977 (513); Pirnay, 1995 (514); Ravussin, 1979 (515); Riddell, 2000 (516); Riddell, 2001 (517); Riddell, 2003 (518); Rowlands, 2005 (519); Saris, 1993 (520); Sutehall, 2022 (521); Yeo, 2005 (522); Noakes, 2025 (120) and S4 (Noakes, 2025 (120)) list studies that have measured the effects of ingesting different amounts and types of CHO during exercise on whole-body CHO and fat oxidation as a result of increasing rates of exogenous CHO oxidation. The essential findings are reproduced in Fig. 16.

Fig. 16A shows that rates of exogenous and whole-body CHO oxidation increase as a linear function of the increasing total CHO ingestion, reaching rates of exogenous CHO oxidation values in excess of 1.5 g/min at ingestion rates greater than 120 g/h. Simultaneously CHO ingestion produces a linear suppression of fat oxidation. Fig. 16B shows that when expressed in kJ/min, the increase in the rate of whole-body CHO oxidation with increasing rates of CHO ingestion is matched by a near isocaloric kJ for kJ reduction in fat oxidation. Fig. 16C shows that the increase in exogenous CHO oxidation substantially exceeds the increased rates of whole-body CHO oxidation so that an increase in exogenous CHO oxidation greater than 1/6 g/min is required to produce a 1-g/min increase in whole-body CHO oxidation. This is not compatible with the concept that increasing exogenous CHO oxidation improves performance solely by increasing rates of whole-body CHO oxidation. Changes in exercise performance with very high rates of exogenous CHO ingestion during exercise have now been reported in 7 studies (Table 9). All studies in which BG concentrations were measured provide evidence that prevention of EIH may have contributed to the beneficial effects of CHO ingestion on exercise performance.

**Table 9.** Performance effects of high rates of carbohydrate ingestion in 8 studies

Study	CHO dosages studied	Exercise test	Outcome	Comment
Smith et al 2010 (176)	0, 15, 30 g/h glucose	120-min cycling exercise at 77% VO_2_max followed by 20-km time trial	Exercise performance significantly improved by 15 g/h glucose but no evidence for dose-response effect >15 g/h	When ingesting placebo, BG concentration fell linearly during 120-min exercise before TT, from 5.3 to 4.5 mmol/L. As a result, individuals ingesting placebo began 20-km TT with lowest (4.5 mM) and falling BG concentration
Smith et al 2013 (260)	0, 10, 20, 30, 40, 50, 60, 70, 80, 90, 100, 110, 120 g/h glucose-fructose-maltodextrin mixture	20-km cycling time trial following 2-h cycling at 95% of workload producing 4 mmol/L blood lactate concentration	Authors proposed curvilinear increase in exercise performance with increasing CHO ingestion. Curvilinear increase emphasized by reducing scale of Y-axis (compare Figs. 1 and 2 in original paper)	BG concentrations not reported; 51 athletes exercised under 13 different conditions at 4 different research sites. Typically each dosage trial involved ∼10 participants. Thus each individual likely completed only 5 of 13 possible dosage combinations
Baur et al 2014 (179)	0, 60, 90 g/h glucose 90 g/h glucose-fructose mixture	30-km cycling time trial following 2-h cycling at 55% W_max._	Although exercise performance improved with CHO ingestion at any dose, there was no evidence for a dose-response effect >60 g/h	BG concentrations fell during 120-min exercise and during 30-km TT only when placebo was ingested. As a result, EIH (3.6 mmol/L) was present in group ingesting placebo at end of TT
Newell et al 2015 (182): Newell et al 2018 (186)	0, 20, 39, 64 g/h glucose mixture	Work-matched cycling time trial (∼30 min at 70% peak power output following 2 h at 95% of lactate threshold	Exercise performance increased with 39- and 64-g/h glucose ingestion but no difference between performance effects produced by glucose ingested at 39 or 60 g/h	When ingesting placebo, participants began 30-min TT with lowest (4.5 mM) and falling BG concentration
King et al 2018 (185)	0, 60, 75 g/h glucose 90, 113 g/h glucose-fructose mixture	30-min TT following 2-h exercise at 77% VO_2_max	Performance improved with 60 g/h vs placebo. No evidence for dose-response effect on performance beyond intake of 60 g/h	When ingesting placebo, participants began 30-min TT with lowest (4.0 mM) and falling BG concentration
King et al 2019 (189)	0, 80, 90, 100 g/h glucose-fructose mixture	30-min TT following 3-h exercise at 60% VO_2_max	All 3 ingestion rates produced superior TT performance vs placebo. Although performance was greatest with intake of 90 g/h, no clear evidence for dose-response with intake from 80 to 100 g/h	When ingesting placebo, participants began 30-min TT with lowest (4.0 mM) and falling BG concentration
Rowe et al 2022 (195)	0 or 90 g/h glucose-fructose or glucose-fructose hydrogel	5 km running time trial following 2-h exercise at 68% VO_2_max	Superior TT performance with 90-g/h CHO ingestion as hydrogel or nonhydrogel	When ingesting placebo, participants began 5-km TT with lowest (4.4 mM) and falling BG concentration
Hearris et al 2022 (264)	0 or 360 g maltodextrin-fructose as liquid, gel, jelly chew, or combination	Exercise capacity test at 150% of lactate threshold, following 180 min at 95% lactate threshold	Superior exercise capacity with 360-g CHO ingestion regardless of delivery method	BG concentrations in placebo trial were not reported. Values rose during 180-min exercise with 360-gCHO ingestion
Podlogar et al 2022 (196)	90 g/h fructose/maltodextrin (2:1 ratio) 120 g/h fructose/maltodextrin (0.8:1 ratio)	No performance trial	No performance trial	Not reported. Fat oxidation profoundly suppressed by CHO ingestion reaching only 0.2 g/min (7.6 kJ/min) after 180-min exercise

Abbreviations: BG, blood glucose; CHO, carbohydrate; EIH, exercise-induced hypoglycemia; TT, time trial; VO_2_max, maximum oxygen consumption.

There are 2 clear conclusions from these studies:

1. They all show that some CHO ingestion during exercise improves performance but fail to establish clear evidence that ingesting more than a minimum effective CHO dose increases performance as a dose-response effect.
2. The most likely explanation for the CHO effect is the prevention of EIH, which was always present in studies in which BG concentrations were measured and in which the placebo was ingested. Indeed, one might suggest that the experimental model required to show an effect of CHO ingestion in all these studies was the same as that originally developed by Coyle et al (99) in 1986 (see Fig. 10), specifically prolonged exercise of a relatively high intensity, especially if it included a period of preexercise fasting to lower liver glycogen content at the start of exercise.

Thus, multiple CHO coingestion increases exogenous and total CHO oxidation, nearly matching reductions in fat oxidation (see Fig. 16B). Yet, increasing CHO ingestion rates show no clear dosage effect on exercise performance (57, 523, 524). Rather “the magnitude of performance change of CHO intake is not affected by the dosage, ergometer used, the type of intake of the CHO ingestion and the type of CHO” (Figures 3a and 3b in (523)). CHO ingestion’s effectiveness increases with exercise duration, highlighting EIH's likely significance as longer exercise raises EIH probability (72, 81, 158-160, 162, 163, 165, 170, 175, 176, 178-180, 185, 187-189, 193, 194, 273, 477, 478). Ramos-Campo et al (523) argued that their study failed to show an expected increase in performance with increasing CHO dose as participants were not trained to ingest CHO at high rates during exercise. However, this is an example of post hoc rationalization (525). Rather, the conclusion could be that their data disproved the accepted hypothesis. The hypothesis that high CHO ingestion rates yield a dose-response performance effect assumes exogenous CHO oxidation perfectly matches ingestion even at high rates (120-144 g/h). However, this analysis establishes that high rates of exogenous CHO ingestion cannot produce equivalently high rates of exogenous CHO oxidation.

Supplementary Table S3 (Noakes, 2025 (120)) includes 12 studies (165, 178, 179, 185, 189, 195, 196, 264, 475, 479, 480, 526) that produced rates of exogenous CHO oxidation at the end of exercise greater than 1.24 g/min (74 g/h), with the highest rates of 1.68 g/min achieved from the ingestion of glucose, fructose, and sucrose at a rate of 144 g/min. Of these studies, those by Hearris et al (264, 475) produced the highest rates of exogenous CHO oxidation during exercise. In the study by Hearris et al (264), participants ingested a 1:0.8 maltodextrin-sucrose (MS) solution at a rate of 120 g/h; in the study by Jentjens et al (475), participants ingested either glucose (G) at a rate of 144 g/h or a mixture of glucose (72 g/h), fructose (36 g/h), and sucrose (36 g/h) (GFS). Our calculations (527) found that total exogenous CHO oxidation from the optimum GFS solution ingested at a rate of 144 g/h during 2 hours of exercise was 137 g or 48% of the total ingestion amount of 288 g. Comparable figures for the MS and G solutions were 112 g (47% oxidized) and 85 g (30% oxidized). This left significant amounts of unoxidized CHO post exercise for all 3 solutions: 151 g (52% unoxidized) for the GFS solution, 128 g (53% unoxidized) for the MS solution, and 203 g (70% unoxidized) for the G solution. Thus, even ingesting CHO at 144 g/h over 2 hours may only increase exogenous CHO oxidation by 68.5 g/h. Supplementary Table S3 (Noakes, 2025 (120)) includes calculations of the extent to which CHO ingested at different doses during exercise will contribute to overall CHO metabolism.

### Summary

Jeukendrup and Jentjens' assumption (465) that increased exogenous CHO oxidation proportionally enhances performance remains unproven. There are 2 possible explanations for this: First, CHO ingested at high rates is not fully oxidized immediately; up to 50% remains unoxidized at exercise termination. Second, as argued in evidence 16 subsequently, CHO's ergogenic effect may stem from preventing depletion of the SGP in the liver and bloodstream, rather than skeletal muscle’s LGP. Prevention of depletion of the SGP requires low rates of CHO ingestion (106, 107). Explanations emphasizing exogenous CHO overlook the universal finding that fat oxidation dominates energy supply late in exercise in all the studies reviewed here (see Figs. 2, 4, 5, 9, 11, and 12 plus Tables 1, 2, 4, 6, and 7) even when CHO is ingested at high rates during exercise.

## Evidence 9: Muscle Glycogen is Not “Spared” During Exercise; Its Use Depends on Initial Muscle and Liver Glycogen Levels, Increasing With High CHO Intake During Exercise

If the oxidation of CHO, specifically muscle glycogen early in exercise, followed by BG oxidation later, is obligatory for sustained exercise performance, then logically the body would attempt to “spare” muscle glycogen use from the start of exercise. Scientists frequently advise athletes to train fat oxidation to “spare” muscle glycogen (528-530). Attempts at nutritional strategies (preexercise CHO-loading, CHO intake during exercise, glucose infusions) to spare muscle glycogen have mostly failed (see Fig. 9) (17, 72, 75, 99, 111, 125, 130, 131, 134, 137, 147, 155, 167, 168, 176, 185, 189, 193, 197, 204, 219, 229, 236, 269, 296, 299, 320, 329, 339, 344, 351, 354, 357, 369, 395, 396, 474, 477, 478, 480-485, 531, 532) with some exceptions (127, 128, 387, 533, 534), including early in exercise (169) or when muscle glycogen concentrations fall below 70 mmol/kg wet weight during exercise lasting more than 2 hours (535). In 1967, Ahlborg et al (17) concluded that: “It thus appears as though the metabolism of added glucose replaces, to a great extent, the fat combustion during work, but cannot replace the energy derived from muscle glycogen” (p. 140).

**Figure 17.**
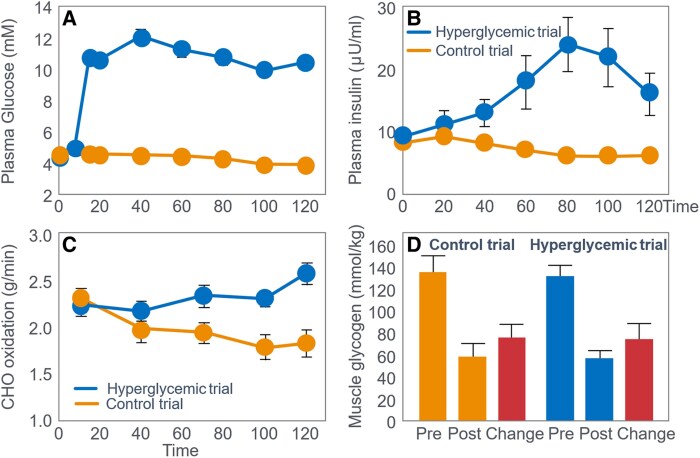
Glucose infused at rates sufficient to produce (A) profound hyperglycemia, (B) elevated plasma insulin conentrations, and (C) increased rates of carbohydrate (CHO) oxidation, failed to influence (D) the rate of muscle glycogen use during prolonged exercise. Reproduced from (486).

Studies of the effects of glucose ingestion during exercise on individual muscle fiber glycogen depletion found that glucose ingestion reduced the rate of muscle glycogenolysis specifically in type 1 muscle fibers (74, 536); another found this effect in type IIa fibers (537). From a study designed to repeat that of Bergstrom et al (4) (see Figs. 1 and 3), Jensen et al (48) concluded that depletion of the intermyofibrillar glycogen stores best explains the development of exercise fatigue and that CHO ingestion specifically spares these stores. However, as in the study by Bergstrom et al (4) (see Fig. 4), participants in the study by Jensen et al (48) also developed marked EIH on the low-CHO preexercise diet. Thus, the authors did not exclude the possibility that EIH rather than depletion of the intermyofibrillar glycogen stores explained the development of fatigue in that study. IV glucose infusion inducing hyperglycemia and high CHO oxidation rates did not reduce muscle glycogen use (Fig. 17) (486).

Thus, paradoxically, fasting (538, 539), intralipid (540-542), or intralipid and heparin infusions (543-546) are the only methods besides low initial glycogen that “spare” muscle glycogen, despite its proposed obligatory role. If muscle glycogen provides the obligatory CHO for oxidation during exercise, why is it not “spared” by exogenous glucose delivered at high rates either via IV or via the gastrointestinal tract? Why is this “obligatory” fuel excessively burned, especially when abundantly available at exercise onset (547)? Why is this obligatory fuel burned in excess even at rest in those eating high-CHO diets (101)? Thus, muscle glycogen likely serves another purpose beyond being obligatory fuel under these conditions (101).

### Summary

The fact that CHO ingestion does not suppress muscle glycogenolysis during exercise, known since 1985, remains largely overlooked (125). Rather, the totality of the evidence presented here strongly suggests that CHO interventions that enhance exercise performance most likely act by delaying the onset of EIH by either reducing liver glycogen use (532) by attenuating neuroendocrine-induced hepatic glycogenolysis induced by inadequate BG levels when glucose levels remain near normoglycemia (see evidence 5), or by increasing liver glycogen supply to blunt EIH by stimulating insulin release and gluconeogenesis subsequent CHO-induced hyperglycemia.

## Evidence 10: High CHO Ingestion Or Infusion Reduces Fat Oxidation, Substantially Increasing Muscle Glycogenolysis

**Figure 18.**
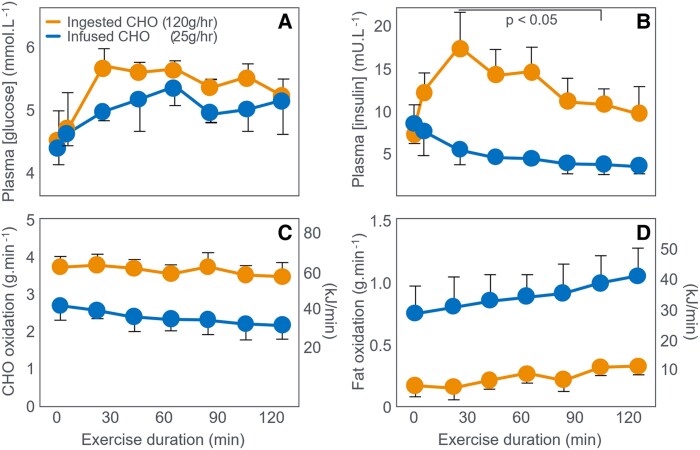
(A) Glucose ingestion (120 g/h) maintained euglycemia during 120 minutes of exercise at 70% VO_2_max as did glucose infused at a rate of 25 g/h. Glucose ingestion increased (B) plasma insulin concentrations and rates of (C) carbohydrate (CHO) oxidation but significantly reduced (D) rates of fat oxidation. Reproduced from (464).

**Figure 19.**
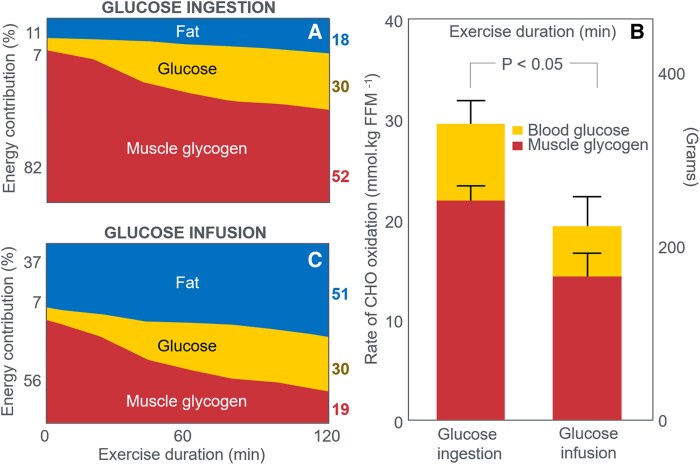
(A) Glucose ingestion at 120 g/h substantially increased the rate and (B) extent of muscle glycogen use during 125 minutes of exercise at 70% VO_2_max, compared to (C) glucose infusion at 25 g/h. This was the result of greatly reduced rates of fat oxidation (infusion: 125 g/h = 51% vs ingestion 25 g/h = 18%). Reproduced from (464).

An overlooked paradox is the differing metabolic effects of CHO ingestion vs infusion (464, 480). Hawley et al (464) showed that the ingestion of 250 g (120 g/h) glucose during 125 minutes of exercise at 70% VO_2_max maintained the BG concentration preventing EIH (Fig. 18A) but raised the blood insulin concentration (Fig. 18B) compared to exercise in which euglycemia was also maintained by glucose infused at a rate of 25 g/h. Ingestion-induced insulin elevations boosted CHO oxidation (Fig. 18C) but reduced fat oxidation (0.3 g/min) to just 30% of lower-rate glucose infusion (1.04 g/min) (Fig. 18D). This fat oxidation inhibition via glucose ingestion substantially increased muscle glycogenolysis (Fig. 19A-19C).

Fig. 19 shows that rates of muscle glycogenolysis were substantially higher when CHO was ingested at 120 g/h compared to when it was infused via IV at 25 g/h.

**Figure 20.**
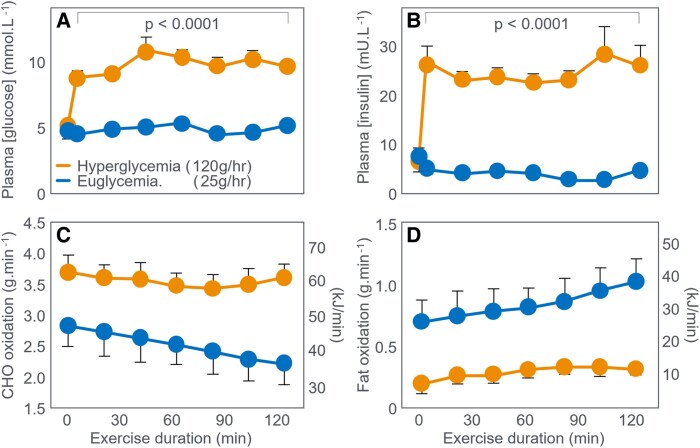
Compared to glucose infusion at a rate of 25 g/h, glucose infusion at 120 g/h produced (A) marked hyperglycemia and (B) hyperinsulinemia, increased the rate of (C) carbohydrate (CHO) oxidation but substantially reduced (D) rates of fat oxidation. Reproduced from (480).

A second study (Figs. 19 and  20), infused 260 g glucose over 125 minutes, inducing hyperglycemia (480) (Fig. 20A); the metabolic response mirrored ingestion of a similar glucose amount (Table 10). However, blood insulin was approximately 2.5 times higher with glucose infusion vs ingestion at comparable rates (∼2 g/min) (compare Figs. 18B and 19B).

**Table 10.** Comparison of metabolic responses to glucose infused at low (0.4 g/min) or high (2.1 g/min) rates or ingested at high (2 g/min) rates during 125 minutes of exercise at 70% maximum oxygen consumption

Metabolic measurement	Glucose ingestion (2 g/min) (413)	Glucose infusion (2.1 g/min) (412)	Glucose infusion (0.4 g/min) (412)	Glucose infusion (0.4 g/min) (413)
Total CHO oxidation (g) in 125 min	450	450	300	300
Rates of CHO oxidation (g/min) at 125 min	3.45	3.60	2.20	2.14
Rates of fat oxidation (g/min) at 125 min	0.32	0.30	1.01	1.04
Total muscle glycogenolysis (g) at 125 min	340	280	250	220
Total glucose oxidation (g) at 125 min	110	170	50	80

Abbreviation: CHO, carbohydrate.

**Figure 21.**
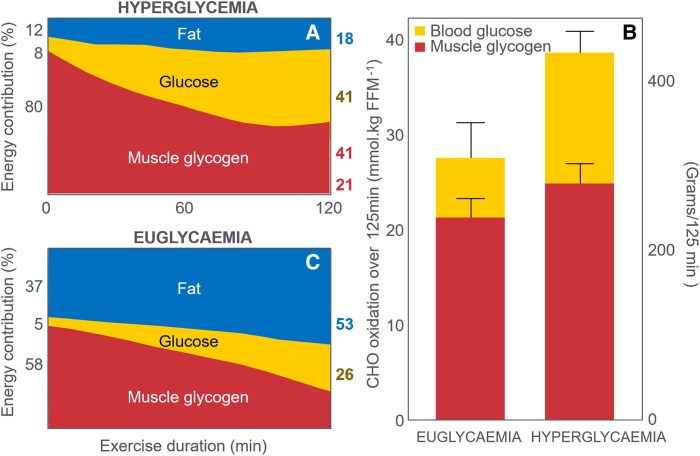
(A) Glucose infusion at 120 g/h substantially increased the rate and (B) extent of muscle glycogen use during 125 minutes of exercise at 70% VO_2_max, compared to (C) glucose infusion at 25 g/h. (A) This was the result of greatly reduced rates of fat oxidation. Reproduced from (464).

Rates of fat, glucose, and muscle glycogen oxidation in the 2 experiments are shown in Fig. 21. As in the previous experiment, high rates of glucose assimilation during exercise elevated blood insulin concentrations, inhibited fat oxidation, and accelerated muscle glycogen use (Fig. 21A-21C).

The overall metabolic responses to high rates of CHO assimilation (120 g/h) were quite similar (see Table 10) whether the glucose was infused or ingested. The key difference was that blood insulin concentrations were higher, as were BG concentrations when the 120 g/h was delivered via IV compared to oral ingestion. This appears in opposition to prior work from Perley et al in 1967 (548), which demonstrated marked elevation in insulin requirements for isocaloric administration of glucose through oral carbohydrates compared to IV infusion. Perley et al explained this effect by elevations in glucagon-like peptide 1 and glucagon inhibitory peptide from oral administration-induced insulin secretion at the pancreas. However, Hawley et al (464) demonstrated marked elevations in circulating glucose levels with IV infusion compared to oral ingestion, which likely explains the elevated insulin levels. These historical comparisons suggest that circulating glucose is a driving factor for insulin release and its subsequent metabolic consequences thereafter.

Perhaps the more interesting findings from these 2 studies are the following:

- Glucose ingested or infused at such high rates substantially **increases** the rates of muscle glycogenolysis (Figs. 18 and 20) as a result of the glucose-induced inhibition of fat oxidation (Figs. 17D and 19D) perhaps because of the increased blood insulin concentration after substantially higher circulation glucose concentrations (Figs. 17B and 19B). Thus, high glucose assimilation accelerates rather than spares muscle glycogen use.
- At these very high rates of glucose infusion or ingestion, as much as 100 g (40%) of the infused glucose could not be accounted for by oxidative removal (480). Therefore, the fate of this excess glucose disappearance is currently unknown. The most likely fate is conversion to triglycerides in the liver (549, 550) especially if fructose constitutes a significant proportion of the CHO load (551). Other studies show that when multiple CHO sources are ingested at very high rates, approximately 27% to 47% of the ingested dose cannot be accounted for by oxidative removal (176, 477, 480).
- Very high CHO ingestion in healthy athletes creates a metabolic profile similar to type 2 diabetes: low fat oxidation and high CHO oxidation (552). This effect appears highly dependent on dietary CHO intake likely influenced by 24-hour blood insulin concentrations (553). Fig. 17B and 17D and 19B and 19D show just how sensitive fat oxidation rates during exercise are to changes in CHO dosage and resulting blood insulin concentrations.

## Evidence 11: Liver Glycogenolysis and BG Oxidation May Be Subject to “Sparing” During Prolonged Exercise

Studies on liver glycogen changes during exercise are limited by the invasive, bleeding-risk nature of liver biopsy. However, it requires no experimental evidence to “prove” that liver glycogen/BG oxidation must be tightly regulated, especially during intense or prolonged exercise. Brooks (106) describes the biological dilemma: “…relative to the meager 4 to 5 g blood glucose pool size in a postabsorptive individual … (exercise-induced) hypoglycemia would occur in less than a minute during hard exercise because blood glucose disposal rate (Rd) could easily exceed glucose production (Ra) from hepatic glycogenolysis and gluconeogenesis.” Whereas higher starting muscle glycogen concentrations increase muscle glycogen use during exercise (71, 79, 80, 291, 391, 397, 554-558), CHO ingestion or infusion during exercise spares liver—but not muscle—glycogen (73, 75, 168, 176, 182, 185, 186, 189, 236, 237, 388, 409, 464, 474, 480, 487, 532, 559). Thus Gonzalez et al (236) conclude that high rates of CHO ingestion (∼102 g/h) during prolonged endurance exercise “prevents the exercise-induced decline in liver glycogen content without modulating muscle glycogen depletion” (p. 1028). Thus, glucose needed to spare hepatic glycogen is likely lower than levels used by Gonzalez et al (236).

Paradoxically and yet to be explained, increasing circulating free fatty acid (and glycerol) concentrations in rats had a much greater glycogen-sparing effect in the liver (543, 545) than in skeletal muscle associated with a greater than 2-fold increase in blood glycerol concentrations for the final 2 hours of a 3-hour exercise bout (545). Exercise time to exhaustion was increased by 1 hour associated with a delayed fall in BG concentrations (545). Heparin infusions in humans spared muscle glycogen (546), raised glycerol more than 10-fold, and increased BG during exercise (546). Conversely, BG oxidation during exercise remains unchanged by higher initial muscle glycogen (291, 391, 477). Glucose infusion during exercise also inhibits hepatic glucose production in proportion to the elevation of the BG concentration (464, 487). This may result from elevated BG directly inhibiting hepatic glucose production (410) via allosteric glycogen phosphorylase regulation (560). Thus, Hawley et al (480) suggest that “glucose oxidation by skeletal muscle is precisely regulated by the prevailing plasma glucose concentration, which in turn, regulates hepatic glucose uptake and release.”

These feedforward and feedback loops help regulate BG homeostasis—increasing glucose removal from the blood (Rd) when BG rises; simultaneously reducing glucose appearance (Ra) derived from the liver.

## Evidence 12: During Prolonged Exercise, Whole-body CHO Oxidation Declines While BG Oxidation Increases, Independent of Intensity. EIH is Likely Without CHO Intake—Especially With Low Liver Glycogen and Limited Gluconeogenesis

RQ declines during prolonged exercise (see Figs. 2, 3, 10, and 12), showing a shift from CHO to fat metabolism (480, 559); the opposite occurs with higher intensity (561, 562). In contrast, BG (as opposed to whole-body CHO) oxidation increases throughout exercise (71, 72, 75, 79, 80, 99, 158, 165, 169, 186, 189, 195, 291, 391, 397, 409, 477, 483, 535, 539, 556, 559, 563-568). Fig. 22 from Hargreaves et al (556) shows how BG Ra and Rd change during exercise depending on initial muscle glycogen levels. Critically, if Rd exceeds Ra, BG concentration must decline.

**Figure 22.**
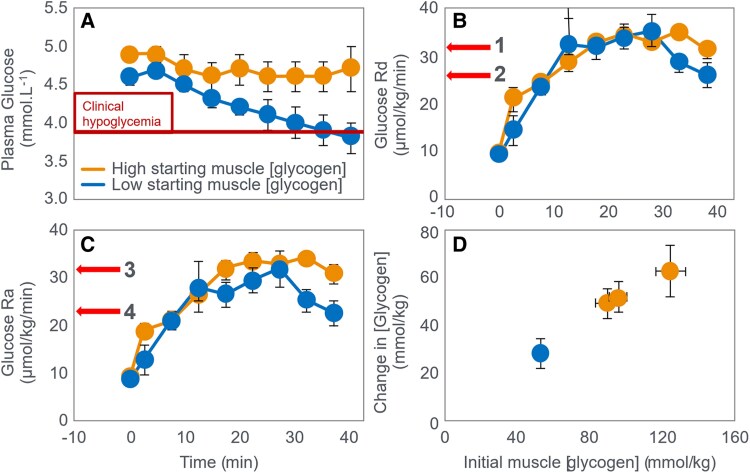
(A) Changes in plasma glucose concentrations; (B) rates of glucose disappearance from the bloodstream (Rd); (C) glucose appearance into the bloodstream (Ra); and (D) muscle glycogen use during exercise that began with either high or low muscle glycogen concentrations. Redrawn from (556). The maximum glucose Rd is 24 g/h (0.4 g/min for a 70-kg individual). Similar values were reported by Trimmer et al (564). Brain glucose Rd at rest comprises approximately 25% of this value; approximately 0.1 g/min (6 g/h) (108).

Fig. 22A shows that BG concentration begins to fall early in exercise after a low-CHO diet when starting liver glycogen concentrations will be low. The rates of Rd (Fig. 22A) initially increase equally and progressively regardless of the starting liver (and muscle) glycogen concentrations (Fig. 22B) but reach higher rates in the CHO-replete state after 30 minutes (arrow 1 in Fig. 22B). In contrast throughout exercise, the rate of Ra (Fig. 22C) in the CHO-depleted state is less than in the CHO-replete state but, importantly, also lower than glucose Rd in the CHO-depleted state. The result is that whereas glucose Ra at 40 minutes (0.42 g/min for 70-kg individual) matches glucose Rd in the CHO-replete state (arrow 3 in Fig. 22C; arrow 1 in Fig. 22B), glucose Ra (0.30 g/min) is substantially less than glucose Rd (0.36 g/min) at the termination of exercise in the CHO-depleted state (arrow 4 in Fig. 22C; arrow 2 in Fig. 22B). This 0.06-g/min gap could reduce the 5-g BG pool to 2.5 g (106, 107) in 42 minutes, dropping BG below 2.5 mmol/L. In this study, elevated Rd after 30 minutes reduced BG by 0.2 mmol/L in the last 10 minutes. This explains why EIH inevitably develops in the CHO-depleted state (82, 83, 106, 107, 176, 409) or during prolonged exercise (>3-4 hours when no CHO is ingested (72, 158-160, 162, 163, 165, 170, 175, 176, 178-180, 185, 187-189, 193, 194, 273, 478).

In the CHO-replete state, higher Rd (compare arrows 1 and 2, panel Fig. 22B) suggests less fat oxidation in certain tissues. Peak glucose Rd is 24 g/h, suggesting CHO intake greater than 24 g/h is likely unnecessary to prevent EIH or maximize oxidation. Fig. 22D shows that, unlike BG Rd (Fig. 22B), which is unaffected by starting liver glycogen, muscle glycogen use is linearly related to its starting concentration, as previously demonstrated. The progressive rise in BG Rd during exercise, even in a CHO-depleted state, appears paradoxical, as it predisposes to EIH without CHO intake. This may reflect an obligatory role in meeting the brain's increasing glucose demand as brain glycogen declines (569-571).

Chronic adaptation to a low-CHO diet reduces total CHO oxidation during exercise by 55%, with a modest decrease in glucose Rd (572), offering additional protection against EIH—similar to adaptations seen with fasted-state training (573). This may reflect partial substitution of obligatory brain glucose use with alternative fuels such as ketones (108, 574), lactate (106), or both, all of which support brain energy metabolism. Evidence supports the brain's preferential use of nonglucose fuels—ketones and lactate—in proportion to their availability, especially during low-glucose states. Cunnane et al in 2016 (575) demonstrated that brain energy demands drive glucose uptake, but ketones are preferentially used when present, as shown by the linear relationship between circulating ketones and cerebral uptake (Fig. 8 from Cunnane, 2016 (575)). Similarly, Siebenmann et al (2021) showed that cerebral lactate uptake during exercise tracks with arterial lactate levels (Siebenmann, 2021 (576)). Notably, ketone utilization occurs even without glucose depletion, as shown by exogenous ketone administration (Cunnane, 2016 (575)), indicating a conserved metabolic flexibility to ensure brain energy needs. Both ketones and lactate cross the blood-brain barrier via Monocarboxylate transporters, which rely on passive diffusion along concentration gradients (Perez-Escuredo, 2016 (577)). Monocarboxylate transporters are upregulated by exercise (Takimoto, 2014 (578)) and fasting (Chasseigneaux, 2024 (579)), enhancing brain access to alternative fuels and protecting against hypoglycemia. Unlike glucose, ketones and lactate do not trigger a counterregulatory response when low. Cahill et al in 1980 (580) showed that elevated ketones (∼1.5 mmol/L) mitigated symptoms of severe insulin-induced hypoglycemia. Prins et al (360) found that despite higher EIH incidence in athletes on low-CHO diets, performance matched that of high-CHO groups, likely due to comparable total circulating brain fuels (glucose + ketones + lactate). These findings suggest that total cerebral fuel availability, rather than glucose alone, is key to preventing a brain energy crisis. Additionally, fasting and low-CHO training may induce liver adaptations that enhance gluconeogenesis, supporting endurance (573 p.244).

Prolonged fasting elicits a similar effect. Knapnik et al (539) showed that a 3.5-day fast reduced muscle glycogenolysis by 58%, glucose Ra and Rd by 40%, and protected against EIH during approximately 120 minutes of exercise at 45% VO_2_max. Likewise, a 23-hour fast did not increase EIH risk during subsequent exercise (581).

## Evidence 13: Falling Liver Glycogen Concentrations Stimulate Adipose Tissue Lipolysis, Increasing Muscle Fat Oxidation, Further Sparing Both BG and Muscle Glycogen Use

**Figure 23.**
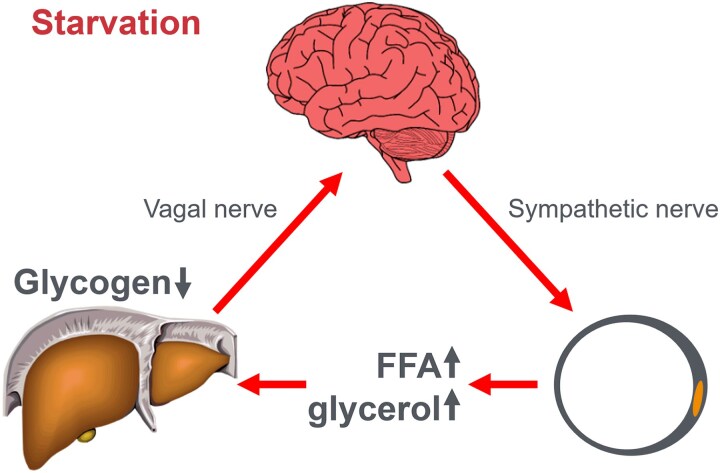
A liver → brain → adipose tissue reflex that increases adipose tissue lipolysis in response to falling liver glycogen concentrations has been described by Izumida et al (582) and Yahagi (583). Whereas increasing blood free fatty acid (FFA) and glycerol concentrations spare muscle glycogen use during exercise (543-546), the effect of increased FFA concentrations on liver glycogen use and the prevention of exercise-induced hypoglycemia is, at least in rats, even greater (543, 545). The mechanism for this effect is currently unknown but could be the result of a glycerol-induced stimulation of hepatic gluconeogenesis. Redrawn from (583).

Falling liver glycogen triggers a liver-brain-adipose reflex that stimulates lipolysis (582, 583), enhancing skeletal muscle fat oxidation and sparing both BG and muscle glycogen (Fig. 23). To our knowledge, no similar mechanism has been described whereby muscle glycogen regulates liver glycogen or BG oxidation (106, 107).

Weltan et al (80) established the role of this liver-brain-adipose reflex in preventing EIH. As they explain: “…glucose oxidation is not increased by reduced muscle glycogen content in subjects with similar blood glucose concentrations; instead, a switch takes place towards lipid oxidation even when plasma glucose concentrations are hyperglycemic. This strengthens the argument in a previous study (79) that this may be a teleological mechanism to compensate for reduced availability of intramuscular CHO without predisposing to hypoglycemia.” This mechanism aligns with the conserved passive GLUT1/3 transporters in the brain and liver and the active GLUT4 transporters in skeletal muscle. The observation that hyperglycemia does not increase BG oxidation in individuals starting exercise with low muscle glycogen (80), alongside tissue-specific passive vs active GLUT transporter patterns, provides strong evidence against the necessity of muscle CHO oxidation during exercise. Instead, human metabolism during prolonged exercise appears geared to delay EIH by increasing skeletal muscle fat oxidation—limiting BG use as liver glycogen declines—and possibly through yet-undefined effects on hepatic glycogenolysis and gluconeogenesis.

## Evidence 14: Preclinical Mechanistic Studies Suggest That EIH, Rather Than Muscle Glycogen Depletion, is the Primary “Exercise Stopper” During Prolonged Exercise

**Figure 24.**
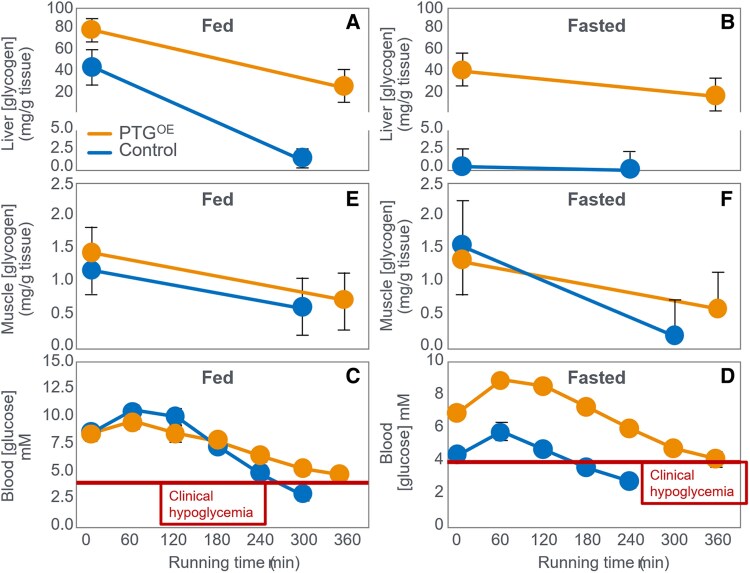
Exercise-induced changes in A and B, liver; E and F, muscle glycogen; and C and D, blood glucose (BG) concentrations in control rats and in rats that overexpress proteins targeting to glycogen PTG^OE^ following preexercise fasting or feeding. Liver glycogen concentrations were elevated under all conditions in PTG^OE^ rats that maintained higher BG concentrations and that exercised for longer, whether fed or fasted. In contrast, muscle concentrations were not different between control and PTG^OE^ rats.

In rats, treatment with the selective gluconeogenesis inhibitor 3-mercaptoicolinic acid (3-MPA) reduced exercise performance by 23% (584). At exhaustion, hepatic glycogen was nearly depleted and BG sharply reduced, while muscle glycogen remained intact in red and white quadriceps fibers. The authors concluded that hepatic gluconeogenesis critically supports BG maintenance during activity, sustaining exercise in controls (584). Conversely, mice overexpressing liver-specific PTG showed enhanced endurance linked to elevated liver glycogen at rest and during exercise in both fed (Fig. 24A) and fasted (Fig. 24B) states (585). They also maintained higher blood glucose during exercise whether fasted (Fig. 24C) or fed (Fig. 24D). Muscle glycogen levels, however, remained unchanged between PTG^OE^ and controls across all conditions (Fig. 24E and 24F). Mice lacking muscle glycogen synthase—and thus unable to synthesize muscle glycogen—show no impairment in exercise capacity, possibly due to preserved liver glycogen synthase activity (586). Similarly, mice with increased preexercise muscle glycogen storage do not exhibit superior performance (587). The authors conclude: “these results identify hepatic glycogen as a key regulator of endurance capacity in mice, an effect that may be exerted through the maintenance of blood glucose levels. Thus, in endurance sports such as marathon running and long-distance cycling, increasing liver glycogen stores should maintain blood glucose and delay the onset of hypoglycemia or “hitting the wall.”

As early as 1975, Baldwin et al (588) showed that prior exercise training better preserves liver than muscle glycogen during subsequent exercise, suggesting liver glycogen sparing is prioritized. They later found liver glycogenesis during recovery occurs 2 to 4 times faster than in skeletal muscle (589).

The assumed necessity of muscle glycogen for skeletal muscle function is challenged by findings that glycogen-depleted type IIb fibers in rats do not fatigue (590); increasing preexercise muscle glycogen does not enhance high-intensity performance (76, 379, 380, 382); and performance is unaffected by prior exercise, a low-CHO diet (382), or their combination (358). Thus, prior exercise may be a stronger determinant of endurance than glycogen availability (591 p.1276). Similarly, high-CHO preexercise diets do not improve strength training performance (592).

## Evidence 15: Maximal Fat Oxidation Rates in High-CHO–Adapted Athletes Underestimate Fat's Contribution to Energy Metabolism, Even During High-intensity Exercise

The emphasis on glycogen and CHO as essential fuels during prolonged or intense exercise is largely based on the crossover point theory, which posits that fat oxidation declines sharply with increasing exercise intensity (87-89, 101, 593). That suggests that above an exercise intensity of approximately 85% VO_2_max, fat oxidation ceases to provide any energy to fuel the exercise. As a result: “CHO-based fuels become the predominant energy source for trained muscle when exercise intensities are >60% of peak oxygen uptake” (90) so that “Clearly at the intensities at which competitive endurance athletes train and race (>70-75% of VO_2_max), the exercising muscles are dependent on carbohydrate for oxidative metabolism (4)” (93). Or “Fat-derived ATP production is designed to provide a “helper fuel” during exercise, with a maximum amount of energy at power outputs at ∼60 to 65% VO_2_max (594)” (92). As a result, “…elite marathon running is nearly 100% CHO-dependent” (91, 93) and that “CHO oxidation (also highly adapted) may be the exclusive source of energy when racing (marathons)” (595) so that the availability of CHO “rather than fat, wins gold medals” (91).

However recent studies challenge this assumption. Prins et al (596) found that on a high-CHO diet, recreational athletes lacked a clear crossover point; even at 40% VO_2_max, more than 60% of energy came from CHO oxidation (Fig. 4D in (596)). In contrast, following adaptation to a high-fat diet for 6 weeks, the crossover point at which more than 50% of their energy came from CHO oxidation increased to 85% VO_2_max (see Fig. 4D in (596)). In contrast, when adapted to the high-CHO diet, only 10% of their energy was provided by fat oxidation at that exercise intensity.

At 80% VO_2_max, fat oxidation rates were 0.97 g/min vs 0.26 g/min after low- vs high-CHO diets, respectively. Two additional studies showed high fat oxidation rates after high-fat diet adaptation during 1.6 km (low-CHO: 0.67 g.fat/min; high-CHO: 0.21 g.fat/min; ∼89%VO_2_max) (31) and 5 km (low-CHO: 0.71 g.fat/min; high-CHO 0.13 g.fat/min; 84%VO_2_max) time trials (TTs) (359). When participants on the high-fat diet performed an interval workout session in which they ran 6 × 800 m (31), some individuals achieved the highest rates of fat oxidation (>2 g/min) yet measured in humans during exercise, and these occurred at an exercise intensity greater than 85%VO_2_max.

These studies likely underestimated true fat oxidation as (i) during progressive exercise, high rates of glycolysis generate an accumulation of H^+^ in the contracting muscle that are transported to the extracellular fluid, which is buffered by [HCO_3_^−^]. This excess (nonoxidative) CO_2_ is excreted through hyperpnoea, elevating the VCO_2_. As a result, indirect calorimetry artifactually elevates respiratory exchange ratio thereby overestimating CHO oxidation and underestimating fat oxidation during high-intensity exercise greater than 70% VO_2_max. (ii) Ketones are present in these athletes at appreciable levels across these studies and the respiratory exchange ratio of acetoacetate (the final step of ketone oxidation) is 1.00 (similar to CHO), whereas the equivalent value for β-hydroxybutyrate is 0.89, higher than fat oxidation values. Burke et al (50, 52, 54) also reported high fat oxidation during progressive exercise, though this was not highlighted. Table 11 shows high rates of fat oxidation (0.79-0.99 g/min) even during exercise at greater than 90% VO_2_max, confirming the findings of Prins and colleagues (31, 101).

**Table 11.** Average rates of fat oxidation during exercise at increasing percentage of maximum oxygen consumption

Study: Burke et al 2016 (50)	Study: Burke et al 2016 (50)	Study: Burke et al 2020 (52)	Study: Burke et al 2020 (52)	Study: Burke et al 2020 (54)	Study: Burke et al 2020 (54)
%VO_2_max	Rate of fat oxidation, g/min	%VO_2_max	Rate of fat oxidation, g/min	%VO_2_max	Rate of fat oxidation, g/min
68	1.50	69	1.40	65	1.40
79	1.49	79	1.36	74	1.30
88	1.39	87	1.22	81	1.25
95	0.79	93	0.99	92	0.95
100	0.25	100	0.45		

Data were extracted from the 3 Race Walker studies (50, 52, 54) using conventional equations.

Abbreviation: %VO_2_max, percentage of maximum oxygen consumption.

**Figure 25.**
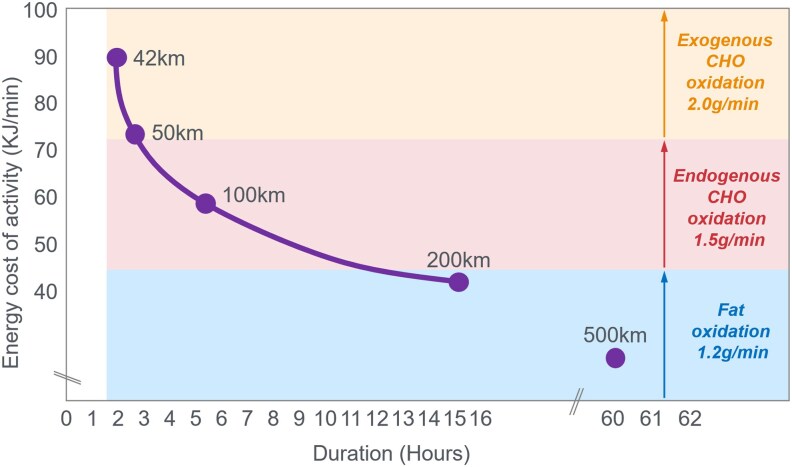
Predicted average rates of energy expenditure (kJ/min) achieved by male athletes setting world records at running distances from 42.2 to 500 km lasting from 2 to 60 hours. The data were calculated from the predicted energy costs of running at different speeds (472) on the assumption that athletes would be no heavier than 60 kg. Included in the figure are predicted reasonable representative rates of fat, endogenous, and exogenous carbohydrate (CHO) oxidation from studies reviewed earlier—(31, 99, 103-105, 359, 360, 367); Figs. 2, 4, 5, 9, 11, and 12, and Tables 2, 3, 6, and 7).

What of the energy requirements of the very best athletes—those who set world records during endurance events? Is it true that “…elite marathon running is nearly 100% CHO-dependent” (90, 91, 93, 595)? Fig. 25 shows predicted energy costs for setting world marathon and ultramarathon records (2-60 hours), and possible supporting fuel sources. Fat oxidation at 1.2 g/min and CHO at 1.5 g/min yield 72 kJ/min—enough for endurance events greater than 3 hours. Only shorter events may require an extra approximately 20 kJ/min from exogenous CHO. According to this prediction, only in events lasting less than 3 hours might there be a need for an additional approximately 20 kJ/min, which could be provided by an exogenous CHO oxidation rate of 1.2 g/min.

**Figure 26.**
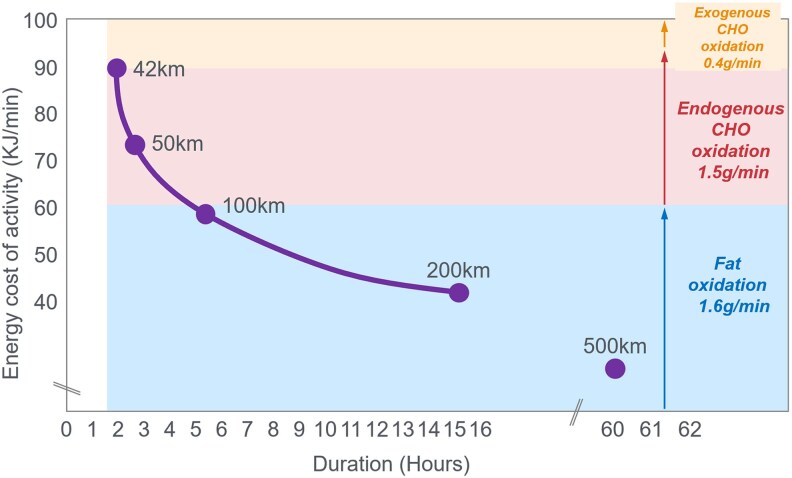
High rates of fat oxidation during high-intensity exercise (85% VO_2_max) (>1.6 g/min; 60 kJ/min) as reported by Prins et al (31), combined with reasonable rates of endogenous carbohydrate (CHO) oxidation (1.5 g/min; 26 kJ/min) would provide most of the energy required to set world running records from 42.2 to 500 km from endogenous fat and CHO storage without the need for ingested CHO oxidation.

Fig. 26 extends this analysis by showing the influence of a slightly higher rate of fat oxidation on the need for additional CHO oxidation to cover the energy requirements when running world records at distances from 42.2 to 500 km.

Though relevant to elite athletes, recreational runners cannot sustain such high intensities. Their performance declines more steeply with increasing exercise duration (470). This is relevant since recreational runners (>99.99% of athletes) often read the same nutritional guidelines that are more specific for elite athletes who represent less than 0.01% of the total exercising population. Eight studies reported ultramarathon runners averaging 5 to 8 km/h (432-434, 445, 446, 462, 463, 471) producing exercise intensities (45%-55% VO_2_max) (434, 444) at which fat oxidation can provide essentially all the required energy, even in CHO-adapted athletes. In one study (434), participants were expending 36 kJ/min during a 24-hour running race, energy that could be fully covered by an exclusive fat oxidation rate of just 0.95 g/min while running at 5.4 km/h (see Figs. 24 and 25). These athletes have little need to ingest CHO at high rates during these ultramarathon races, in contrast to what the modern dietary guidelines currently promote (see Evidence 7).

### Summary

High fat oxidation rates at greater than 85% VO_2_max challenge the belief that glycogen is an obligatory fuel and that only exogenous CHO can sustain performance when it is depleted. Still, debate over glycogen's role as an obligatory exercise fuel continues (30, 597-599).

## Evidence 16. Endurance Performance After 6 Weeks of High- or Low-CHO Diets Showed No Difference. But 10 gCHO/h During Exercise on Both Diets Boosted Performance by 12% to 20%, Linked to Elimination of EIH

To differentiate between the influence of the SGP and LGPs on prolonged exercise performance, Prins et al (360) manipulated preexercise muscle glycogen content by habituating athletes to either high- (380 gCHO/d) or low- (40 gCHO/d) CHO diets for 6 weeks prior to exercise testing. After a 15-hour preexercise fast that followed either diet, participants then performed prolonged exercise to exhaustion when ingesting either a sweet-tasting CHO-free placebo or CHO at a rate of 10 g/h designed to prevent depletion of the SGP without having a major effect on whole-body metabolism. This (small) amount of CHO was chosen to test the hypothesis that the ergogenic effects of CHO ingestion during exercise results from the prevention of EIH rather than from the provision of a source of obligatory CHO to glycogen-depleted skeletal muscles, in line with the interpretation of the results shown in Table 9. Whereas there was no difference in exercise performance following habituation to either the low- or high-CHO diets, performance increased by 12% to 20% when individuals ingested just enough CHO to prevent EIH without affecting whole-body fuel metabolism to any great extent.

Another recently completed study came to the same conclusion. Carpenter et al (322) studied a group of recreational athletes who had been following a self-prescribed low-CHO ketogenic diet for a mean duration of 25 months. Participants performed a 16.1-km cycling TT under 4 conditions—(i) placebo ingestion for 2 days before the TT together with placebo ingestion 30 minutes before exercise (P); (ii) CHO ingestion for 2 days before the TT with placebo ingestion 30 minutes before exercise (SHORT); (iii) placebo ingestion for 2 days before the TT with CHO ingestion 30 minutes before exercise (ACUTE); and (iv) CHO ingestion for 2 days before exercise followed by CHO ingestion 30 minutes before exercise (COMBO). CHO intake for 2 days before exercise (SHORT condition) did not improve performance vs placebo (P). Thus, boosting LGP glycogen before exercise had no effect—exactly as predicted by the evidence presented herein. CHO intake 30 minutes before exercise (ACUTE and COMBO) also improved performance and maintained BG levels during exercise. Accordingly, these 2 studies (322, 360), perhaps the first of their kind to attempt intentionally to differentiate between the contributions of the SGP during prolonged exercise performance, proved unequivocally that the role of the SGP is dominant since it determines whether a falling BG concentration will develop during exercise. They also clarify the curious findings of a 1993 study (147) that the authors themselves could not explain, perhaps because it conflicted with the dominating doctrine that the pr-exercise muscle glycogen content is the key determinant of performance during prolonged exercise.

**Figure 27.**
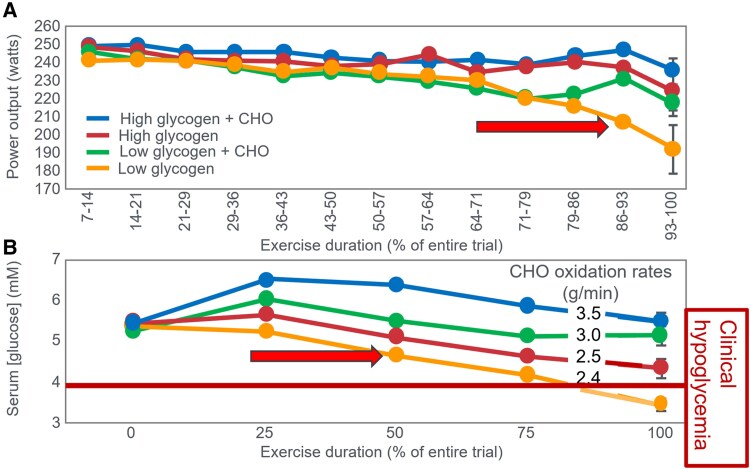
Changes in A, power outputs and B, blood glucose (BG) concentrations in individuals who followed different preexercise dietary and exercise manipulations to produce high and low preexercise muscle glycogen concentrations (high or low glycogen) followed by ingestion of glucose (+CHO) or placebo during exercise to achieve different BG concentrations. The horizontal arrow in the B indicates that BG concentrations in the low glycogen intervention without carbohydrate (CHO) ingestion during exercise begin to fall after 50% of the time trial (TT) had been completed, whereas power output falls precipitously only after 86% of that TT had been completed (arrow in B). Rates of CHO oxidation in the 4 different trials are superimposed on the appropriate lines in the bottom panel. The increase or maintained power output in the final 30% of the TT in the low glycogen + CHO trial could not be explained by relatively small difference in rates of CHO oxidation (overlaid in B). Reproduced from reference (147).

As tested 31 years later by Prins et al (359, 360), those authors compared the effects of CHO- and placebo-feeding during exercise in individuals who began exercise with either normal or dietary- and exercise-induced reductions in their preexercise muscle glycogen concentrations (Fig. 27). Relevant to this discussion is that these interventions would also have altered the preexercise liver glycogen concentrations in the SGP (23-25).

The surprising finding was that, provided they ingested CHO during exercise, the exercise performance of individuals who began 2 hours of exercise with low muscle (and liver) glycogen content in the low glycogen + CHO trial was only marginally worse (−3%) than when they began exercise with normal muscle (and liver) glycogen content in the high glycogen trial (Fig. 27A). Furthermore, CHO ingestion during exercise in the high glycogen + CHO trial did not produce a superior performance compared to the high glycogen trial (see Fig. 27A). Participants adopted a different anticipatory pacing strategy (600) in the low glycogen + CHO compared to the low glycogen without CHO trial (see Fig. 27A). Only after 70% of the TT had been completed did the low glycogen + CHO group increase and maintain a higher power output compared to the low glycogen trial (without CHO ingestion). The period of increasing power output in the final 30% of that TT was associated with the avoidance of hypoglycemia (Fig. 27B). Perhaps this anticipatory increase in power output near the end of exercise—the endspurt phenomenon (600, 601)—might have happened earlier if athletes in the low glycogen + CHO trial had been exposed to additional bouts of exercise habituation while eating a low-CHO diet. The authors noted with surprise that: “Although carbohydrate feeding allowed subjects to maintain their work output under conditions of relatively low muscle glycogen (concentrations), they (CHO ingestion during exercise) appear to provide little ergogenic benefit during 120 minutes of strenuous exercise performed after a glycogen-super-compensation protocol” (p. 3003) so that “when pre-exercise muscle *(and liver*—*present authors' addition*) glycogen levels are elevated, carbohydrate feedings provide little ergogenic effect during a 120-min self-paced exercise bout” (p. 3004). Muscle glycogen use was approximately 56% higher when starting with normal vs low levels and was not reduced by CHO intake (Table 1 in (147)). Therefore, better performance in high glycogen trials was not due to glycogen sparing. Notably, individuals with low starting glycogen reached lower terminal levels, implying early termination in high glycogen trials may not reflect true depletion thresholds. Instead, Fig. 27B shows CHO intake's key benefit was preventing progressive BG decline—especially in those starting with low muscle (and likely low liver) glycogen, which led to clinical hypoglycemia in the low glycogen + placebo trial.

In hindsight, the 1993 findings by Widrick et al likely reflect higher preexercise CHO intake raising glucose both in LGP and SGP (147). But exercise did not significantly deplete the SGP, so BG did not drop in the high glycogen + placebo group. They began with enough SGP glucose to avoid EIH, so extra CHO during exercise gave no benefit. Conversely, the low glycogen intervention reduced both LGP and SGP glucose. Thus, performance effects are better explained by reduced SGP rather than LGP.

The findings from these 3 studies are therefore in line with the findings of this comprehensive review, which is that the protection of the SGP during exercise best explains the well-established ergogenic effects of CHO ingested during exercise (112, 113, 602).

## Summary and Conclusions

A series of research publications by Scandinavian scientists Olaf Boje, Erik Hohwu Christensen, and Ole Hansen immediately preceding World War II established that CHO ingestion during exercise prolongs exercise performance. They proposed that, acting by a central neural mechanism, CHO ingestion reverses EIH, allowing exercise to continue without the risk of glycopenic brain damage. The introduction of the skeletal muscle needle-biopsy technique in the 1960s, also by Scandinavian scientists, produced an iconic 1967 study that argued for a causal relationship between (i) starting muscle glycogen content and the duration for which prolonged submaximal exercise could be sustained and (ii) the development of fatigue with the onset of muscle glycogen depletion. Since then, generations of sports scientists have concluded (i) that human skeletal muscle has an obligatory requirement to oxidize CHO, preferably muscle glycogen, during prolonged exercise especially when the exercise is more intense, and (ii) that fatigue develops whenever those muscle glycogen stores are depleted. Overlooked in that iconic 1967 study was evidence that profound EIH was also present at exhaustion, especially in individuals eating the most CHO-restricted diets. Hence that study could not exclude the possibility that EIH had contributed to the fatigue. Importantly, whereas CHO ingestion even in small amounts can reverse EIH within minutes by increasing the glucose content of the SGP in the liver and circulating blood, no intervention can selectively and rapidly refill any glucose deficit in the large skeletal muscle glucose pool. Thus, it is not currently possible to determine the precise contribution made by the large skeletal muscle glucose pool to exercise fatigue in humans. Without such evidence, this review demonstrates that the initial exercise-stopper in prolonged exercise is an inevitable reduction in the glucose content of the SGP in those who do not ingest CHO during prolonged exercise and in whom the rate of hepatic gluconeogenesis is inadequate to match a progressive increase in BG oxidation during prolonged exercise. This mismatch leads to a progressive reduction in the BG concentration and, ultimately, to EIH. Then, as first proposed by Boje, Christensen, and Hansen, the falling BG concentration activates a brain-directed control that constrains the exercise performance specifically to prevent the development of glycopenic brain damage.

The evidence for this different interpretation is presented in 16 separate topics:

1. The mechanism of depletion of the large skeletal muscle glucose pool causes fatigue remains hypothetical. The usual explanation is the TAT, which postulates that muscle glycogen depletion during exercise causes an inevitable “energy crisis.” But this is implausible since a developing “energy crisis” must produce skeletal muscle ATP depletion leading ultimately to muscle rigor, not whole-body fatigue. But this does not happen. A more probable explanation is that exercise fatigue is a brain-control mechanism designed to cause exercise termination before irreversible energy depletion occurs in the exercising skeletal muscles.
2. The iconic 1967 Scandinavian study failed to acknowledge that individuals eating CHO-restricted diets terminated exercise, not with low muscle glycogen content alone, but also with marked EIH.
3. Early studies by Boje, Christensen, and Hansen showing that CHO ingestion could reverse exhaustion in individuals during prolonged exercise concluded that “Fatigue must be regarded as a hypoglycemic symptom of cerebral origin.”
4. Series of carefully conducted studies in the 1980s concluded that Boje, Christensen, and Hansen's interpretation was incorrect, postulating rather that CHO ingestion prolongs exercise, not by preventing or reversing EIH, but by providing an obligatory (exogenous) source of CHO for muscles that have become glycogen depleted during prolonged exercise. However, none of these new studies provided definitive evidence to support this replacement hypothesis, which currently remains unproven and largely untested with little or no experimental support.
5. Eighty-eight percent of studies that reported a clear benefit on exercise performance of CHO ingestion before or during exercise also found that BG concentrations fell during exercise in the control (placebo) group. This is significant since few if any of these studies were actively studying a falling BG concentration as an important factor causing exercise fatigue.
6. The neural mechanisms by which falling BG concentrations limit the recruitment of skeletal muscle motor units to prevent the risk of glycopenic brain damage is well described. The biological effect of hypoglycemia on exercise performance must be considered relative to an individual's baseline glucose levels and the magnitude of glucose reduction, as counterregulatory responses and symptoms vary by person, can occur subperceptually, and may manifest at higher glucose levels in those with chronically elevated glycemia. Consequently, the “drop” in BG relative to a prior value has important biological consequences in exercise performance beyond the absolute value of BG.
7. Increasing the amount of CHO ingested during exercise increases the rate of exogenous CHO oxidation. According to the novel replacement theory, this additional source of obligatory CHO should produce an easily detectable dose-dependent increase in exercise performance. However, studies of progressively higher rates of CHO ingestion during prolonged exercise fail to show serial dose-dependent improvements in exercise performance. Rather, the lowest CHO dose that is tested usually produces ergogenic effects equivalent to those produced by much higher rates of CHO ingestion. This effect is most logically explained by CHO acting to prevent depletion of the small glucose pool in the blood and liver, rather than by depletion of the large glucose pool in skeletal muscle.
8. The novel replacement hypothesis has produced expert guidelines encouraging athletes to ingest up to 2-g CHO/min during prolonged exercise to prevent depletion of the LGP. Illogically, these guidelines are also promoted to athletes participating in exercise lasting more than 3 hours. Yet, prolonged strenuous exercise (eg, ultramarathon races) are often completed at exercise intensities (60%-75% VO_2_max) at which intensities fat oxidation should be able to provide a substantial proportion of the required energy, especially as rates of fat oxidation increase with progressive muscle glycogen depletion (see also topic 15).
9. Muscle glycogen is not “spared” during prolonged exercise as would be expected if the LGP is the cardinal driver of exercise performance. Instead, the rate of muscle glycogen use during exercise is set by the muscle glycogen concentration at the start of exercise. Paradoxically high rates of CHO ingestion during exercise increase muscle glycogenolysis.
10. This is because high rates of CHO ingestion or infusion during exercise reduce rates of fat oxidation. This would explain the increased rates of muscle glycogenolysis with high rates of CHO ingestion or infusion during exercise. In effect, muscle glycogen determines its rate of use during exercise by setting the rate at which the major alternate fuel, fat, is oxidized. This effect appears to be hormonally regulated.
11. In contrast, CHO ingestion during exercise reduces or, at high rates of intake, completely suppresses liver glycogenolysis. This effect is the result of control mechanisms aimed at maintaining a stable BG concentration, which is considered the principal aim of human metabolism.
12. During prolonged exercise, whole-body CHO oxidation decreases, while BG oxidation progressively increases, regardless of intensity. Consequently, EIH is probable without CHO intake, particularly when starting with low liver glycogen and limited hepatic gluconeogenesis capacity.
13. Falling liver glycogen concentrations stimulate adipose tissue lipolysis, increasing muscle fat oxidation, further sparing both BG and muscle glycogen use.
14. Rats that overexpress the PTG have elevated liver glycogen content at rest and during both exercise and fasting. They also have greater resistance to EIH and superior exercise performance. Rats with a greater capacity to store muscle glycogen do not have superior exercise performance. Nor do rats unable to synthesize muscle glycogen have impaired exercise performance. The authors conclude that: “…these results identify hepatic glycogen as a key regulator of endurance performance in mice, an effect that may be exerted by the maintenance of blood glucose concentrations.”
15. Recent studies establish that athletes chronically adapted to low-CHO diets achieve the highest rates of fat oxidation yet measured in humans (>1.5 g/min) even when exercising at more than 85% of their VO_2_max. This challenges the existence of the exercise crossover point, which holds that CHO oxidation increases and fat oxidation decreases with increasing exercise intensity so that above approximately 85% VO_2_max only CHO is oxidized. Rather, this finding suggests that fat oxidation fuels exercise even during high-intensity exercise. This in turn challenges the concept that CHO is an obligatory fuel both for high-intensity and prolonged exercise.
16. A recent study found that the performance of athletes habituated for 6 weeks to either high- (380 gCHO/d) or low- (40 gCHO/d) CHO diets was not different during prolonged submaximal exercise, despite low-CHO diet having lower glycogen and CHO oxidation levels. However, ingesting 10-gCHO/h during exercise increased exercise performance by 12% to 20% while eliminating EIH. As glycogen and whole-body CHO were lower on low-CHO diet across multiple analyses, yet performance was maintained, this directly challenges glycogen and whole-body CHO oxidation and instead suggests EIH is a central determinant of exercise performance.

These data demonstrate that the main benefit of CHO ingestion before or during exercise is to prevent the development of EIH, which appears inevitable if the exercise is prolonged for more than 2 to 3 hours and is undertaken by individuals who are unable to increase hepatic gluconeogenesis sufficiently. Accordingly, this has important implications for advice on the value of habitual high- or low-CHO diets for athletes as well as the amount of CHO athletes should be encouraged to ingest during exercise to maximize performance. The data reviewed herein present the novel interpretation that nutritional strategies to maximize performance during prolonged exercise should be geared to maintaining the SGP during exercise rather than filling (or overfilling) the LGP before exercise. The present evidence indicates that this can be achieved by ingesting relatively small amounts of CHO (∼10 g/h) during exercise.

## Data Availability

Supplemental and Meta data can be found at https://zenodo.org/records/16085777 (Noakes, 2025 (120)) and https://thenoakesfoundation.org/. Some or all remaining datasets generated during and/or analyzed during the current study are not publicly available but are available from the corresponding author on reasonable request.

## Abbreviations

ATP: adenosine triphosphate
BG: blood glucose
CHO: carbohydrate
EIH: exercise-induced hypoglycemia
IV: intravenous
LGP: large glucose pool
MS: maltodextrin-sucrose
PTG: protein targeting to glycogen
Ra: blood glucose appearance
Rd: blood glucose disappearance
RQ: respiratory quotient
SGP: small glucose pool
SMMUR: skeletal muscle motor unit recruitment
TAT: the anaplerotic theory
TT: time trial
VO_2_max: maximum oxygen consumption
